# Structural and Functional Role of INI1 and LEDGF in the HIV-1 Preintegration Complex

**DOI:** 10.1371/journal.pone.0060734

**Published:** 2013-04-11

**Authors:** Benoit Maillot, Nicolas Lévy, Sylvia Eiler, Corinne Crucifix, Florence Granger, Ludovic Richert, Pascal Didier, Julien Godet, Karine Pradeau-Aubreton, Stéphane Emiliani, Alexis Nazabal, Paul Lesbats, Vincent Parissi, Yves Mely, Dino Moras, Patrick Schultz, Marc Ruff

**Affiliations:** 1 Institut de Génétique et de Biologie Moléculaire et Cellulaire, Département de Biologie Structurale intégrative, Université de Strasbourg, U596 INSERM, UMR7104 CNRS, Illkirch, France; 2 Laboratoire de Biophotonique et Pharmacologie, UMR 7213 CNRS, UDS, Faculté de Pharmacie, Illkirch, France; 3 Institut Cochin, Université Paris Descartes, CNRS (UMR8104), INSERM (U567), Paris, France; 4 CovalX AG, Zürich-Schlieren, Switzerland; 5 Laboratoire de Microbiologie Fondamentale et Pathogénicité, CNRS (UMR5234), Université de Bordeaux 2, Bordeaux, France; University of South Carolina School of Medicine, United States of America

## Abstract

Integration of the HIV-1 cDNA into the human genome is catalyzed by the viral integrase (IN) protein. Several studies have shown the importance of cellular cofactors that interact with integrase and affect viral integration and infectivity. In this study, we produced a stable complex between HIV-1 integrase, viral U5 DNA, the cellular cofactor LEDGF/p75 and the integrase binding domain of INI1 (INI1-IBD), a subunit of the SWI/SNF chromatin remodeling factor. The stoichiometry of the IN/LEDGF/INI1-IBD/DNA complex components was found to be 4/2/2/2 by mass spectrometry and Fluorescence Correlation Spectroscopy. Functional assays showed that INI1-IBD inhibits the 3′ processing reaction but does not interfere with specific viral DNA binding. Integration assays demonstrate that INI1-IBD decreases the amount of integration events but inhibits by-product formation such as donor/donor or linear full site integration molecules. Cryo-electron microscopy locates INI1-IBD within the cellular DNA binding site of the IN/LEDGF complex, constraining the highly flexible integrase in a stable conformation. Taken together, our results suggest that INI1 could stabilize the PIC in the host cell, by maintaining integrase in a stable constrained conformation which prevents non-specific interactions and auto integration on the route to its integration site within nucleosomes, while LEDGF organizes and stabilizes an active integrase tetramer suitable for specific vDNA integration. Moreover, our results provide the basis for a novel type of integrase inhibitor (conformational inhibitor) representing a potential new strategy for use in human therapy.

## Introduction

During the early events of viral replication the RNA genome is converted into its cDNA copy which then, upon interaction with cellular and viral proteins, generates the pre-integration complex (PIC). Cellular trafficking along the microtubule network transports the PIC to the nuclear envelope. The lentivirus subfamily PICs exhibit karyophilic properties which enable them to enter the nucleus through the nuclear pore. To establish a productive infection, the viral cDNA must subsequently be integrated into the host genome by the integrase protein (IN), which is a permanent component of the virion and the PIC. IN performs several important steps in the life cycle of retroviruses. It was shown to be involved in several steps of HIV-1 replication, such as uncoating [1], reverse transcription [2], nuclear import [3], chromatin targeting [4] and integration [5]. Viral components such as IN cannot perform all these functions by themselves and need to recruit host cell proteins to efficiently carry out the different activities. The molecular details and temporal sequence of these processes, and particularly the role of cellular co-factors, remain largely unknown.

The IN enzyme consists of three structural and functional domains, namely the N-terminal zinc binding domain (residues 1–50), the central catalytic core domain (CCD; residues 50–212) containing the D, D, E triad that coordinates divalent ions and the C-terminal domain (residues 213–288). A systematic study of mutants in the catalytic core identified a mutation (F185K) which greatly increases its solubility [6]. This mutant was used for high resolution structural studies. Several partial structures of HIV-1 IN have been solved, namely the CCD domain alone [7]–[10], as well as the CCD domain combined with the C-terminal domain [11] or the N-terminal domain [12] and finally, the CCD in complex with the IN binding domain of LEDGF [13]. Structures of IN from other retroviruses have also been solved [14]. In these structures, the catalytic core is organized into a highly conserved dimer except for the IN encoded by the Rous associated virus type-1 [15], whereas the position of the N-terminal and C-terminal domains relative to the catalytic core domain is extremely variable (**Fig. S1**). Recently, the structures of two functional integration units have been solved, namely the crystallographic structure of the Prototype Foamy Virus (PFV) IN/DNA complex [16] and the cryo Electron Microscopy (cryo-EM) structure of the HIV-1 IN/LEDGF/DNA complex [17]. To validate the comparison between the two structures we solved the EM structure of the PFV IN tetramer (**Fig. S2 and methods S1**). The X-Ray structure of the PFV IN could be readily fitted in the envelope showing that the overall arrangement of the IN domain does not depend of the method used (EM and X-ray). Both structures showed that the functional unit is composed of an IN tetramer. The comparison of the two structures revealed a different organization of the monomers in the tetrameric unit (**Fig. S3**). Moreover, most of the residues showed to be important for DNA binding and/or 3′processing in the HIV-1 integrase model constructed using the PFV IN structure [18] are also in interaction with DNA in our EM model [17].Taken together, the data reveal a high flexibility in the linkers between the IN domains as well as in their oligomeric organization. This inherent flexibility explains the propensity of IN to interact with multiple partners and to intervene in numerous biological functions by exposing and reshaping interaction surfaces [19]–[21]. The final arrangement of the domain is probably strongly dependent of the interaction with protein co-factors and IN function in the infected cell (microtubule migration, nuclear internalization, chromatin targeting and integration). Several cellular co-factors have been shown to be important for HIV-1 infection and to interact with HIV-1 IN [22]–[26]. Among them, the INtegrase Interactor protein 1 (INI1) which is a homolog of yeast SNF5, the core component of the SWI/SNF chromatin remodeling complex [27], and the Lens Epithelium-Derived Growth Factor (LEDGF) [28], a transcriptional co-activator. The function of LEDGF in HIV-1 infection is to target IN to chromosomes of infected cells [29]. Its expression is required for proviral integration and subsequent production of HIV-1 virions [30]. At the structural level, the interaction with LEDGF was shown to produce an IN active form by maintaining a stable HIV-1 IN tetramer [17].

INI1 was the first protein shown to interact with IN [27]. The 385 residue long INI1, contains a C-terminal SNF5 homology domain with 3 highly conserved sequence motifs: repeat 1 and 2 and a coiled-coil motif (**Fig. S4**). Repeat 1 was found to be necessary and sufficient to bind to IN [31]. The role of INI1 in the HIV-1 replication cycle remains controversial, but it has been clearly established that it acts both on the early and late stages of viral infection, probably by distinct mechanisms. In the late stage, INI1 may facilitate proviral transcription by enhancing Tat function [32]–[36]. Indeed, INI1 could act as a regulating factor to initiate one of two mutually exclusive transcription programs after integration, namely post-integration latency or high-level, Tat-dependent gene expression [37]. It has also been shown that over-expression of the INI1 integrase binding domain in the cell inhibits HIV-1 assembly by specifically binding to viral gag-pol protein [38]. Finally, INI1 was shown to be incorporated in mature virions with a stoichiometry of 1 INI1 for 2 IN molecules [39] and to incorporate SAP18 HDAC complex into virions [40]. INI1 has been shown to both increase [38], [41], [42] and inhibit [43] viral replication. *In vitro* experiments on reconstituted nucleosomes have demonstrated that purified SWI/SNF complexes stimulate viral DNA integration by restoring the DNA accessibility to IN via nucleosome remodeling [41].

In order to clarify the INI1 - mediated inhibition and/or activation functions in the early stage of HIV-1 infection, we analyzed the structure-function relationships of a quaternary complex comprising the full length wild type HIV-1 IN, the full length wild-type LEDGF, the INI1 IN binding domain (174–289) (INI1-IBD) and viral U5 DNA. We first showed that the IN/LEDGF complex performs concerted integration more efficiently than isolated IN molecules and with a higher fidelity regarding the structure of the integrated DNA expected for HIV-1. In the presence of INI1-IBD, integration events are decreased whereas the formation of integration by-products (donor/donor and linear FSI) are strongly reduced when compared to IN alone or to the IN/LEDGF complex. Fluorescence anisotropy measurements showed that the dissociation constants of IN/LEDGF and IN/LEDGF/INI1-IBD for U5 vDNA remain in the same order of magnitude, indicating that viral DNA interaction is not impaired by the presence of INI1-IBD. Analysis of the 3′ processing activity demonstrated that INI1-IBD, when bound to the IN/LEDGF complex, inhibits the 3′ processing reaction. Mass spectrometry and FCS analysis showed that 2 INI1-IBD, 2 LEDGF and 2 U5 vDNA molecules interact tightly with an IN tetramer. Finally, the structural analysis by cryo-EM of the IN/LEDGF/DNA [17] and INLEDGF/INI1-IBD/DNA complexes revealed the binding sites of LEDGF, INI1-IBD and U5 vDNA on the IN tetramer. INI1-IBD interacts with IN on the opposite side of the LEDGF binding site and within the target DNA interaction region, locking IN in a stable constrained conformation. Taken together, our data show that INI-IBD steadies the highly flexible IN protein in a compact stable conformation. This suggests that the role of the full length INI1 in the early stage of infection could be to stabilize IN and prevents its inter-domain flexibility, thus preventing non-specific interaction and auto integration on the route to its specific nucleosome targets, while LEDGF organizes and stabilizes an active IN tetramer suitable for specific vDNA integration.

## Results

### Purification and Characterization of the IN/LEDGF/INI1-IBD Complex

In order to study the effect of INI1 on the structure and function of IN we intended to add a fragment of INI1 onto the previously characterized, highly soluble and active IN/LEDGF complex. The method used to produce the complexes yields a soluble, homogeneous and active entity. This is in sharp contrast with most of the earlier work on full length integrase which is known to be prone to aggregation. A soluble fragment of INI1 containing the *bona-fide* integrase binding domain was identified and produced using a structural genomic strategy. The amino acid sequence of INI1 was analyzed by a combination of programs, including multiple alignment [44] and various prediction tools [45] to define domain limits. A total of 16 fragments were cloned in fusion with 3 different affinity tags (MBP, GST, HIS) and were tested for expression and solubility. The INI1 fragment spanning residue 174 to 289 in fusion with 6 histidines was selected (**Fig. S4**).

Full length IN, full length LEDGF and the INI1 (174–289) (INI1-IBD) fragment were purified separately and solubilized using high salt and CHAPS. The IN/LEDGF/INI1-IBD complex was formed upon removal of the solubilizing agents by dialysis and was purified to homogeneity by affinity chromatography and gel filtration which showed a sharp and symmetric peak (**Fig. S5A**). The stoichiometry of the partners was determined by High-Mass MALDI ToF mass spectrometry analysis [46]. Control experiments identified the mass of the three components: IN (MH^+^ = 32.8 kDa), LEDGF (MH^+^ = 60.4 kDa) and His_6_-INI1-IBD (MH^+^ = 17.2 kDa) (**Fig. S5B**). In a second step, the purified complex was chemically cross-linked prior to mass spectrometry. Trace amount of several protein and complexes were detected: [INI1-IBD] (MH^+^ = 17.8 kDa), [IN·IN] (MH^+^ = 64.8 kDa) and [LEDGF·LEDGF] (MH^+^ = 122.6 kDa,), but the mass of the major species corresponded to [4IN·2LEDGF·2INI1-IBD] (MH^+^ = 283.4 kDa) (**Fig. S5C**). Higher molecular weight complexes in the range between 500–1000 kDa were not detected, indicating that the complexes did not aggregate. These experiments show that a stable complex is formed between the previously characterized [4IN·2LEDGF] complex which incorporates 2 molecules of INI1-IBD.

### In vitro DNA Binding Properties

#### Evidence for the binding of two viral U5 DNA duplexes to the IN/LEDGF complex

To determine the number of viral U5 DNA duplexes bound to the IN/LEDGF complex, we used Fluorescence Correlation Spectroscopy (FCS) with viral U5 DNA duplex (40 bp) modified on one of its 5′ ends by Texas red (TXR). In the absence of IN/LEGDF, the autocorrelation function *G(τ)* of the U5 vDNA-TXR duplexes indicated a single diffusion time (*D_1_*) of 97+/−3 µm^2^•s^−1^ (**Fig. S6A**), fully consistent with the diffusion of a DNA duplex of 26 kDa [47].The distribution of brightness (**Fig. S6B**), obtained from a large number of measurements (n ≈ 60), was nearly mono-disperse with a median value of 0.77+/−0.07 kHz per U5 vDNA-TXR duplex. Addition of IN/LEDGF to the U5 vDNA-TXR duplex solution shifted the autocorrelation curve to longer diffusion times (**Fig. S6C**), indicating an increase in the molecular weight of the diffusing species, in line with an interaction of U5 vDNA-TXR duplex with IN/LEDGF. Moreover, the observed increase in the Y-axis intercept of the autocorrelation curve, which is inversely proportional to the number of diffusing species, indicated a decrease in the total number of diffusing species (**Fig. S6A**). This suggests that more than one U5 vDNA-TXR duplex interacts with each IN/LEDGF complex. According to the binding experiments (see below), a fraction of the U5 vDNA-TXR duplexes in solution is likely to be not bound to the IN/LEDGF complexes in the FCS conditions. Therefore, to take into account the presence of both free and bound vDNA-TXR molecules, the autocorrelation curves were fitted by a two-population model (**Eq. 2 in methods S1**). To limit the number of variables in the fitting process, the value of the correlation time τ_D1_ for the free molecules was fixed, using the aforementioned value obtained with U5 vDNA-TXR duplex alone. From the fit, the value of the diffusion constant of the U5 vDNA-TXR/IN/LEDGF complexes (*D_2_*) was found to be 51+/−0.2 µm^2^·s^−1^, suggesting that the molecular weight of the complexes is about 300 kDa. Moreover, the ratio of brightness between the complex of U5 vDNA-TXR duplex with IN/LEDGF and free U5 vDNA-TXR duplex (B2/B1) was found to be 1.96+/−0.62, further indicating that the IN/LEDGF complex binds two U5 vDNA-TXR duplexes. Finally, the ratio (N2/N1) (ratio between the number of U5 vDNA-TXR/IN/LEDGF complexes and the number of free U5 vDNA-TXR duplexes) was 1.30+/−0.07, a value very close to that (1.25) calculated from the Kd value determined by fluorescence anisotropy (see below). Taken together, these results show that two U5 vDNA duplexes are bound to one IN/LEDGF complex. Moreover this experiment demonstrates that the IN/LEDGF complex is homogenous and does not aggregate in the presence of DNA.

#### Determination of binding constants by fluorescence anisotropy

**Figure 1:**
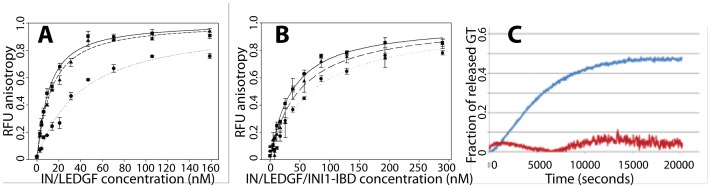
In vitro functional tests. **A–B:** Determination by fluorescence anisotropy of the dissociation constant of viral DNA to IN/LEGDF and IN/LEDGF/INI1-IBD. **A**: Titration curve of viral DNA by the IN/LEDGF complex in the absence (square, continuous line) or in the presence of an excess of non-fluorescent viral DNA (circles, dotted line) and non-fluorescent non-specific DNA (triangles, dashed line). **B**: Same as A for the IN/LEDGF/INI1-IBD complex. Lines correspond to the fits of equation 8 in **methods S1** to the data. **C**: 3′ processing reaction followed by fluorescence anisotropy. The fraction of released GT as a function of time is shown in blue for the IN/LEDGF complex and in red for the IN/LEDGF/INI1-IBD complex.

The binding constants of the viral U5 DNA duplex for the IN/LEDGF and IN/LEDGF/INI1-IBD complexes were determined by fluorescence anisotropy. The viral U5 DNA duplex (40 bp) of the same sequence as for the FCS experiments was modified at one of its 5′ends by 6-Carboxyfluorescein (6FAM). As expected, an increase in the fluorescence anisotropy was observed upon addition of increasing concentrations of protein to a fixed concentration of DNA. The dissociation constant (Kd) was calculated using the Scatchard equation rewritten to fit the anisotropy data [48] as described in the **methods S1**. A stoichiometry of 2 U5 vDNA duplexes per IN/LEGDF or IN/LEDGF/INI1-IBD complex was assumed, based on the FCS experiments. The Kd values found for the IN/LEDGF and IN/LEDGF/INI1-IBD complexes are respectively 10.6+/−0.5 nM and 35+/−4 nM (**Fig. 1A, B**). These values are similar to those found in previous studies [**49**].To assess the specificity of the binding sites for U5 vDNA duplex, competition experiments with an excess of non-fluorescent specific and non-specific DNA duplexes were performed. While the latter induced no shift in the titration curve, excess of non-fluorescent specific U5 vDNA duplex was found to shift the binding curve, in line with a competition of fluorescent and non-fluorescent specific U5 vDNA duplex for the binding sites. This indicates the specificity of both IN/LEDGF and IN/LEDGF/INI1-IBD complexes for U5 vDNA duplexes. Taken together these data indicate that the IN/LEDGF complexes, with and without INI1-IBD, specifically bind the U5 DNA complexes with binding constants that differ only by a factor of three.

### Influence of INI1-IBD and LEDGF on the 3′ Processing Reaction

To investigate the 3′-processing reaction catalyzed by the IN/LEDGF and IN/LEDGF/INI1-IBD complexes, we used HIV-1 U5 viral DNA duplex with the same sequence as for the FCS and fluorescence anisotropy experiments, but labeled at one of its 3′ends by 6-FAM. The 3′ processing reaction with this DNA duplex releases a fluorescent GT-FAM dinucleotide, which results in a decrease of the fluorescence anisotropy [50]. The release of GT-FAM was monitored as a function of time for the IN/LEDGF and IN/LEDGF/INI1-IBD complexes (**Fig. 1C**). The results clearly show that the 3′ processing reaction is fully inhibited in the ternary complex (IN/LEDGF/INI1-IBD), indicating that INI1-IBD does not affect DNA binding but protects the 3′ends of the viral DNA from endonucleotidic cleavage by IN. This result has been confirmed using a gel based 3′ processing assay (**Fig. S7**).

### Influence of INI1-IBD and LEDGF on the Integration Reaction

**Figure 2:**
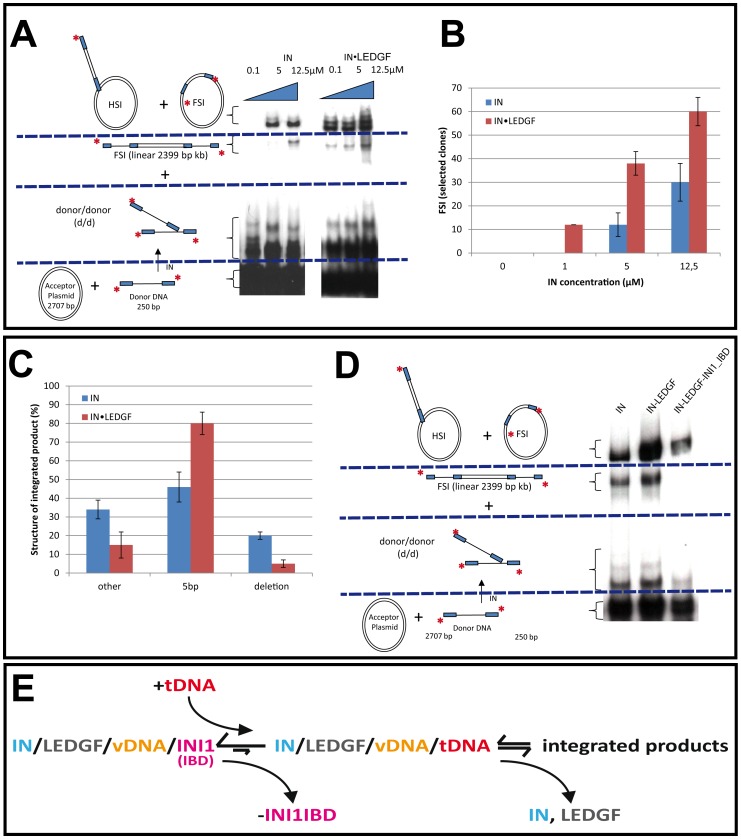
Comparative analysis of the in vitro concerted integration catalyzed by HIV-1 IN, HIV-1 IN/LEDGF and HIV-1 IN/LEDGF/INI1-IBD complex. **A.** In vitro integration profiles of HIV-1 IN and HIV-1 IN/LEDGF complexes. Concerted integration assay was performed using 0.1 to 12.5µM IN or IN/LEDGF complex (concentration are normalized to IN monomers), 150 ng of acceptor plasmid, 15 ng of radioactively labelled and processed donor DNA. The reaction products were loaded on 1% agarose gel. The position and the structure of the different products obtained after half-site (HSI), full-site (FSI) and donor/donor (d/d) integration are reported. **B.** Full site integration activity. The circular FSI products obtained in (A) were quantified by cloning in bacteria and reported as the number of ampicillin-, kanamycin- and tetracycline-resistant selected clones [62]. **C.** Structure of the integration products. One hundred circular FSI integration products were isolated in condition where the concerted integration assay was performed with 12.5 µM of IN or IN/LEDGF complexes and were sequenced as reported in the materials and methods section. The number of correct 5 bp duplication, deletions and other structures are reported as percentages. All values reported are the mean ± standard deviation (error bars) of three independent sets of experiments. **D.** Effect of INI1 on the integration profile. The assay was performed and reported as in (A) using 12.5µM IN, IN/LEDGF or IN/LEDGF/INI1-IBD complex (in IN monomers). A quantification of the gel showing the strong decrease in linear fsi and dd product is shown in **figure S8**. **E** Proposed equilibrium between the different complexes involved. In the presence of tDNA, INI1 is slowly displaced from the IN/LEDGF/INI1-IBD/vDNA complex and the integration reaction can occur to produce specific integration products.

The concerted integration reaction performed under standard conditions demonstrates that the recombinant IN/LEDGF complex performs this reaction in a highly efficient way. Half Site Integration (HSI), Full Site Integration (FSI) and donor-donor (d/d) integration products were detected by gel electrophoresis and showed that the global integration efficiency was higher for the IN/LEDGF complex than for isolated IN molecules (**Fig. 2A**). Specific cloning and quantification of the circular FSI products attested that the IN/LEDGF complex catalyses 2 to 10 times more concerted integration events than isolated IN molecules (**Fig. 2B**). In addition the structure of the integrated viral DNA was analyzed by sequencing the cloned circular FSI products. The sequences clearly show that the integration reaction catalyzed by the IN/LEDGF complex is closer to the expected physiological reaction than IN alone since it produced two times more HIV-1-specific 5 bp staggered cuts of the target DNA (**Fig. 2C**). To investigate the effect of INI1 on the integration reaction, a comparative analysis of the reaction products was performed with the IN, IN/LEDGF or IN/LEDGF/INI1-IBD complexes. In the presence of INI1-IBD, a reduction of the integration events was observed as well as an inhibition of by-product formation such as d/d or linear FSI molecules (**Fig. 2D**). The integration activity detected in the presence of INI1-IBD results from a competitive displacement of INI1-IBD by tDNA as shown in **Fig. 2E**. In vitro, this equilibrium is probably strongly displaced towards the quaternary IN/LEDGF/INI1-IBD/vDNA complex and would be slowly displaced towards the integrated products as the IN/LEDGF/vDNA complex is used and INI1-IBD displaced by the tDNA. Altogether, in the presence of INI1-IBD, integration occurs with reduced kinetics compared to IN alone or to the IN/LEDGF complex, but with strongly reduced by-products formation (donor/donor or linear FSI).

### Cryo-electron Microscopy Structure of the IN/LEDGF/INI1-IBD/vDNA Complex

To better understand the mechanism by which INI1-IBD inhibits the 3′ processing reaction and by-products formation in the integration reaction, we determined the structure of the IN/LEDGF/INI1-IBD/DNA complex by a two-step electron microscopy strategy. We first imaged the purified IN/LEDGF/INI1-IBD complexes after negative staining in order to determine a low resolution model from which the position of INI1-IBD can be deduced by comparison with previous data on IN/LEDGF. Then we imaged frozen hydrated, unstained IN/LEDGF/INI1-IBD/vDNA particles to solve a higher resolution structure of the full complex.

**Figure 3:**
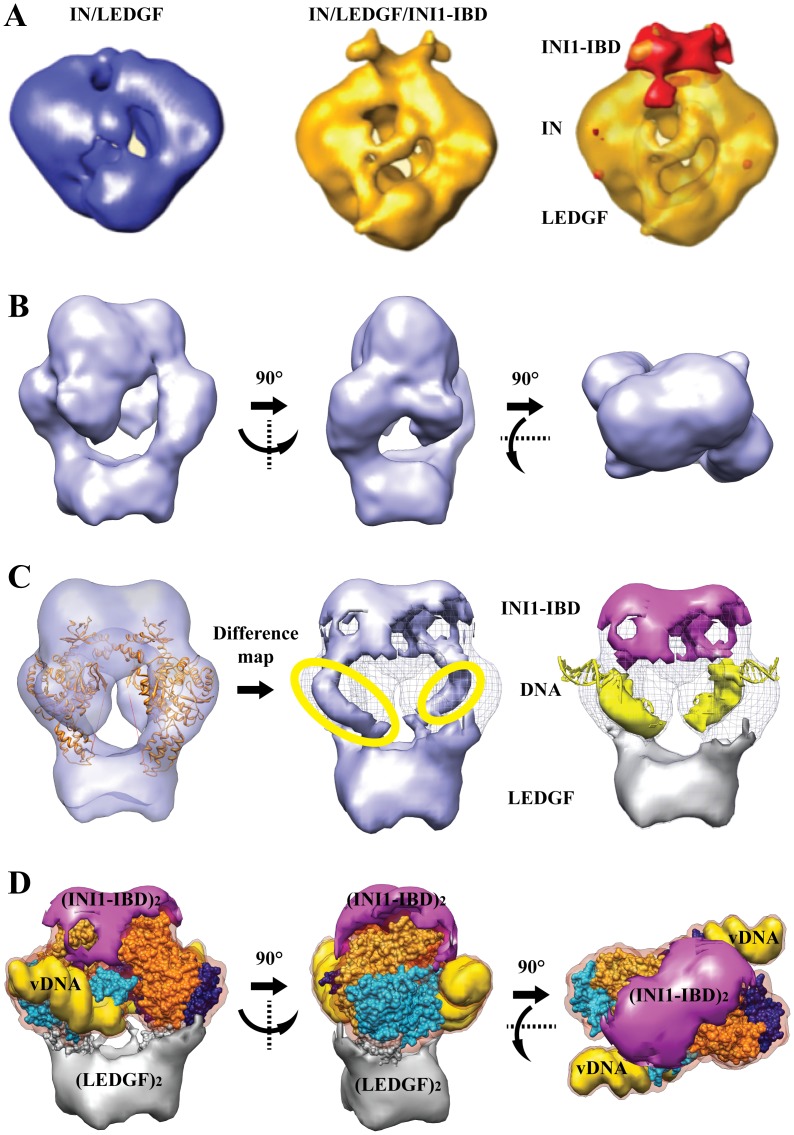
Cryo-EM structure. **A**. Negatively stained structures of the IN/LEDGF (blue) and IN/LEDGF/INI1-IBD (gold) complexes. The difference between the two maps representing the position of INI1-IBD is in red. **B**. Cryo-EM structure of the IN/LEDGF/INI1-IBD/DNA complex represented by three perpendicular views. **C**. Difference map calculated by subtracting the density corresponding to the fitted atomic models of IN and LEDGF from the IN/LEDGF/INI1-IBD/DNA cryo-EM map. The differences, corresponding respectively to INI1-IBD, DNA and LEDGF, are represented in violet, yellow and grey. **D**. Three perpendicular views of the IN/LEDGF/INI1-IBD/DNA complex: INI1-IBD is in violet, LEDGF in grey and viral DNA in yellow. The fitted IN model is represented as molecular surface. For both IN dimers, the two monomers are in blue and gold. The two IN dimers (light and dark) are related by a twofold symmetry.

The image analysis of negatively stained single particles by correlation averaging methods revealed views about 14–15 nm in size, several of which showed a clear 2-fold symmetry. Particles of similar size and symmetry were previously observed for the IN/LEDGF complex [17], thus indicating that the addition of INI1 does not modify the oligomerization state of the IN-containing complexes. A 3-D model of the IN/LEDGF/INI1-IBD complex was determined and the difference map with the IN/LEDGF complex showed a positive difference in the region where the target DNA was shown to interact [17] (**Fig. 3A**).

The IN/LEDGF/INI1-IBD complex was then incubated with a tenfold molar excess of the 21-mer U5-substrate. The particles were homogeneous in size and uniformly distributed on the grid. A dataset of 12781 images of unstained, frozen hydrated IN/LEDGF/INI1-IBD/vDNA complexes was recorded and analyzed independently from any previous model. A 3-D model was built using the angular reconstitution protocol [51], followed by a search for a two-fold axis to impose a symmetry that was observed in all IN/LEDGF complexes analyzed so far. The volume enclosed by the 3-D model shown in **Fig. 3B** is consistent with the stoichiometry of 4 IN, 2 LEDGF and 2 INI1-IBD molecules (∼283 kDa) determined by mass spectrometry. A resolution of 18 Å was obtained according to the Rosenthal and Henderson criteria [52] (**Fig. S9**).

**Figure 4:**
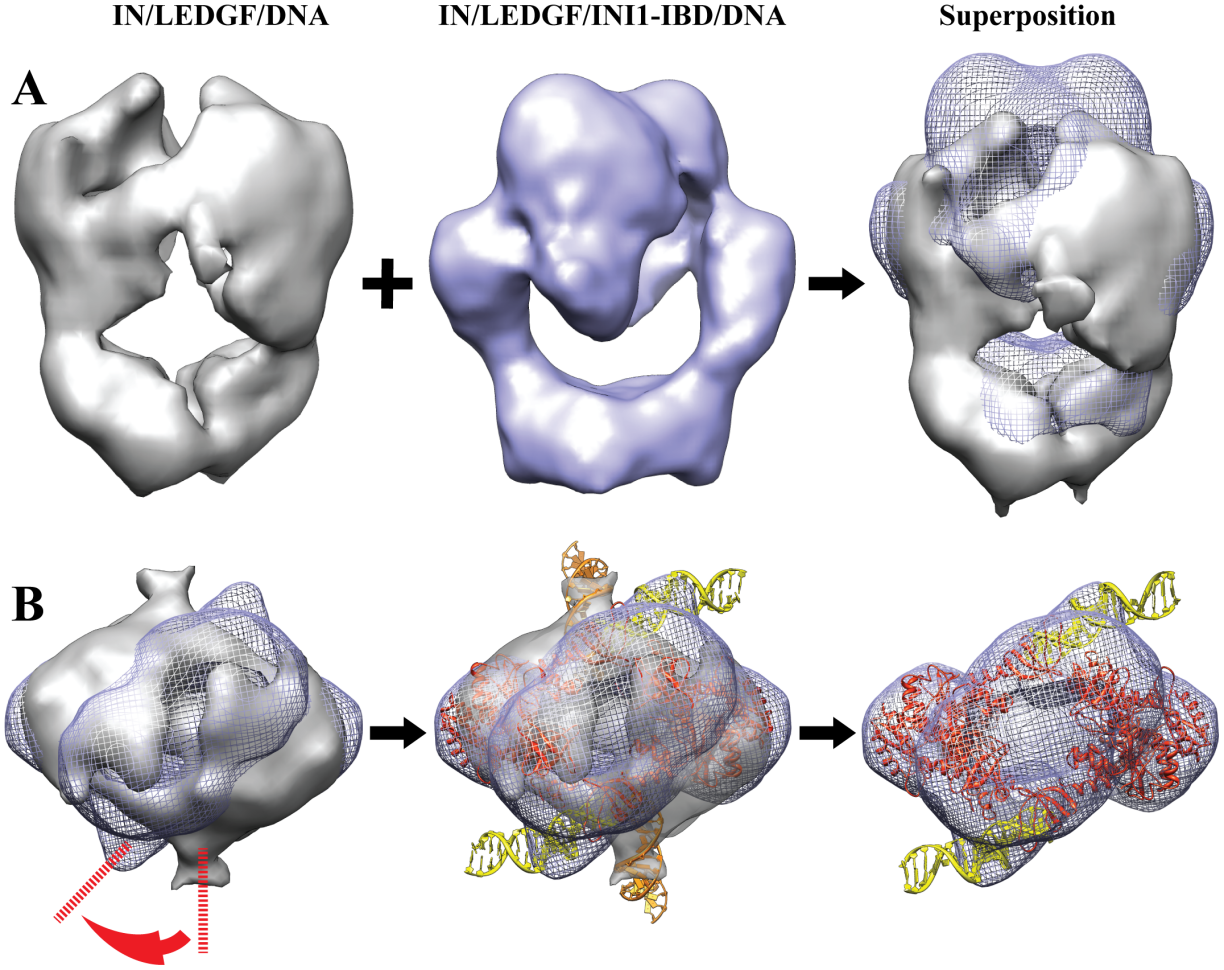
Superimposition of the IN/LEDGF/DNA and IN/LEDGF/INI1-IBD/DNA structures. **A:** Superimposition of the IN/LEDGF/DNA (grey) and IN/LEDGF/INI1-IBD/DNA (blue) structures. **B:** DNA fitting. The spikes in the two structures unambigously revealed the DNA position. The DNA (yellow) is fitted in the IN/LEDGF/INI1-IBD/DNA map (mesh representation) with a 40° rotation from its position in the IN/LEDGF/DNA map (surface representation).

**Figure 5:**
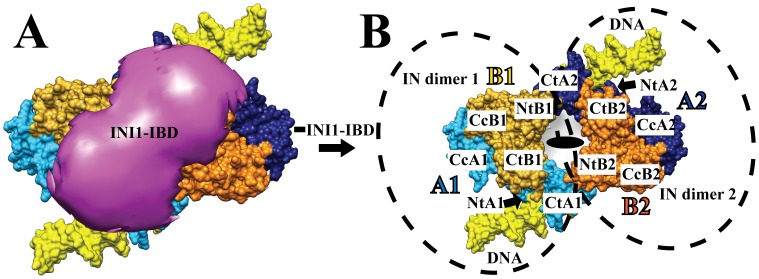
Topology of the IN tetramer. **A:** View from the INI1-IBD side of the complex. **B:** View of the IN tetramer after removing the INI1-IBD density. The two IN dimers (1 and 2) are related by a twofold axis perpendicular to the sheet. Each IN monomer (A and B) is represented in a different color (blue and gold). The two monomers related by the two fold symmetry are represented in light and dark color (light blue for A1 and dark blue for A2, light gold for B1 and dark gold for B2). The positions of the IN domains are indicated: Nt for the N-terminal domain, Cc for the catalytic core and Ct for the C-terminal domain.

The maps of the IN/LEGDF/DNA [17] and the IN/LEGDF/INI1-IBD/vDNA complexes could be readily superimposed (**Fig. 4A**). The existing atomic structures of IN (catalytic core, N-and C-terminal domains) and of the IN-Binding Domain of LEDGF were pre-positioned as determined previously in the IN/LEGDF/DNA model. They were further refined by normal mode flexible fitting (NMFF) and structure idealization. The fitting parameters were chosen in a way that does not modify the fold of the protein domains, as described in the **methods S1**. The position of the IN tetramer was found to be unchanged upon addition of INI1-IBD. A density difference map was then calculated between the cryo-EM map and the fitted atomic structures, in order to reveal the positions of INI1-IBD, LEDGF and DNA (**Fig. 3C**). The difference map shows three groups of additional densities, corresponding to the IN interaction partners (INI1-IBD, LEDGF, vDNA). A saddle-shaped density is observed on the top of the IN dimers (**pink density in** **Fig. 3C**), which corresponds to INI1-IBD since its position is identical to the one found in the absence of DNA (**Fig. 3A**). A second group of additional densities is detected within the viral DNA binding sites, corresponding to bound DNA molecules. The difference map reveals rod-like structures consistent with the size and shape of DNA molecules (**yellow densities in** **Fig. 3C**). In the presence of INI1-IBD, two U5 vDNA duplexes could be fitted in the electron density map. Interestingly, U5 viral DNA duplexes are rotated by about 40° in the INI1-IBD containing complex, as compared to the IN/LEDGF/DNA strand transfer model [17] (**Fig. 4B**). The third group of additional densities is located at the bottom of the structure and corresponds to the LEDGF protein which contributes to stabilize the complex (**grey density in** **Fig. 3C**). The fitting of the atomic models into the IN/LEDGF/INI1-IBD/vDNA map was then further refined in order to reveal more clearly the positions of LEDGF, INI1-IBD and vDNA (**Fig. 3D**). The atomic structures fitted into the cryo-EM map showed that the complex contains 4 IN, 2 LEDGF, 2 INI1-IBD and 2 U5 vDNA molecules, confirming the mass spectrometry and FCS data. The structure of the complex is organized around two asymmetric IN dimers. The first dimer, formed by two monomers (A1, B1), shows different positioning of their respective N and C terminus and is related to the second dimer (A2, B2) by a twofold symmetry (**Fig. 5**). The INI1-IBD dimer interacts on the top of the IN tetramer and contacts mainly the N and C-termini of the two IN B monomers (NtB1, NtB2, CtB1, CtB2). A small lateral extension of INI1-IBD reaches the C-terminus of the two A monomers (CtA1, CtA2), in close proximity with the viral U5 DNA duplex (**Fig. 5**).

## Discussion

HIV-1 IN is the platform protein present in all steps of the retroviral cycle involving the preintegration complex (PIC). IN forms the structural core of the PIC and is most probably involved in PIC migration along microtubules [53], transfer to the nucleus [54], as well as chromatin targeting [55] and integration. Comparison of known structures of retroviral INs shows the high conformational flexibility of its different domains, depending on the virus type and the presence of interacting host proteins. This conformational flexibility explains the ability of IN to interact with multiple partners and to exert multiple biological functions. To gain further insight into the regulation of IN functions by host factors, we investigated the structures and interactions of IN with the cellular LEDGF and INI1-IBD proteins, as well as their impact on IN activities.

The IN/LEDGF complex was established to be composed of 4 IN and 2 LEDGF molecules [17] but little information was available on the binding of viral DNA. Based on a brightness analysis, FCS shows that two U5 viral DNA (vDNA) duplexes can bind to this complex. Furthermore, the diffusion constant measured by FCS for the IN/LEDGF/vDNA complex (51 µm^2^.s^-1^) is consistent with the theoretical diffusion constant of the IN_4_-LEDGF_2_-vDNA_2_ complex (37.2 µm^2^.s^-1^), calculated from its dimensions (15.5 nm×14 nm×10 nm) determined by EM. Thus, FCS confirms that IN_4_-LEDGF_2_-vDNA_2_ is the major complex in solution. The addition of INI1-IBD to IN/LEDGF led to a stable IN/LEDGF/INI1-IBD complex which indicates that both cellular proteins can bind simultaneously to IN. By further adding U5 vDNA duplex, an IN/LEDGF/INI1-IBD/vDNA complex was formed thus demonstrating that neither host factor interferes with vDNA binding. Fluorescence anisotropy confirms that U5 vDNA duplexes bind specifically to both IN/LEDGF and IN/LEDGF/INI1-IBD complexes, with affinities of 11 and 35 nM, respectively. Thus, INI1-IBD only weakly affects the binding of vDNA to the complex.

The Cryo-EM structure of the IN/LEDGF/INI1-IBD/vDNA complex fully agrees with the stoichiometry of 4 IN, 2 LEDGF, 2 INI1-IBD and 2 vDNA molecules determined by FCS and mass spectrometry and furthermore reveals the interaction sites of INI1-IBD, LEDGF and vDNA with IN (**Fig. 5** **,** **6**). INI1-IBD interacts mainly with the C-terminal domains of two IN monomers (CtA and CtB) and with the N-terminal domain of monomer B (NtB). In this position INI1-IBD does not sterically interfere with the DNA binding site of IN which appears occupied in the 3-D model as predicted by the binding studies.

**Figure 6:**
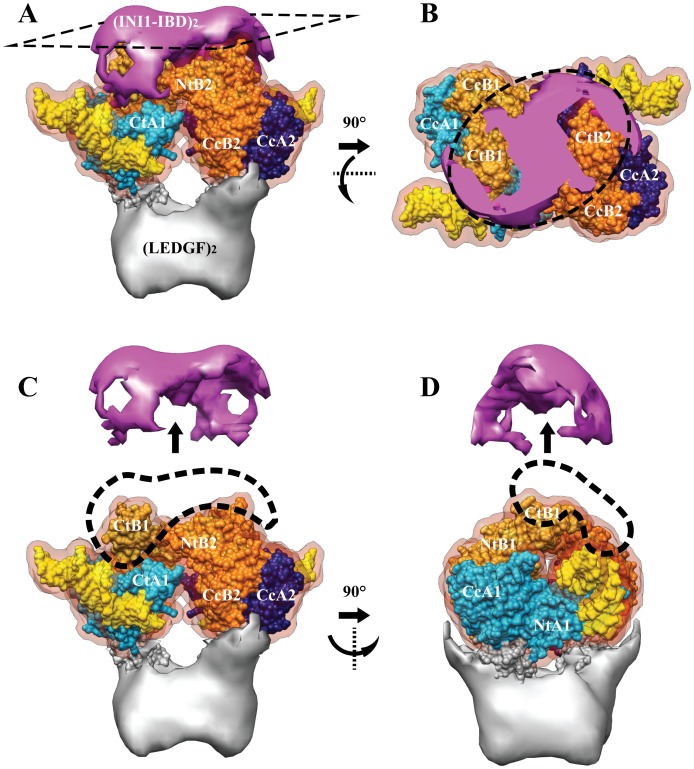
IN/LEDGF/INI1-IBD/DNA structure. **A**. INI1-IBD (pink) sits on the top of the complex and interacts with the C and N termini of the two IN monomers B. An extension of INI1-IBD interacts with the C-terminus of the two monomers A. **B**. A 90° view sliced as shown by the dotted square in figure A. **C and D**: two 90° views showing clearly the IN interacting domains with INI1.

**Figure 7:**
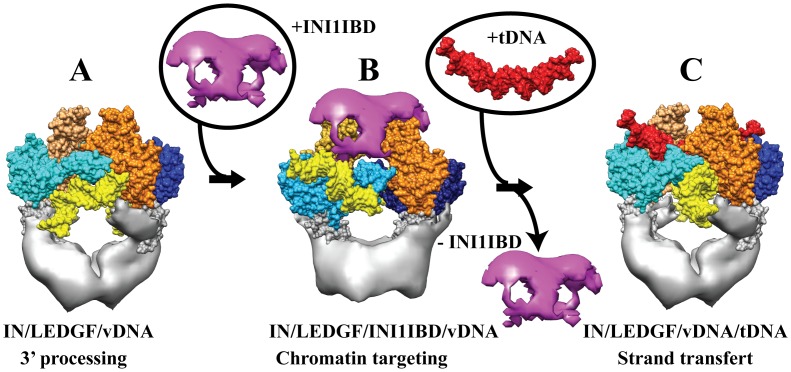
Structure-function analysis. **A**. IN/LEDGF/vDNA structure in the 3′ processing conformation. **B**. In the IN/LEDGF/INI1-IBD/vDNA complex, INI1-IBD caps the surface of the complex and blocks IN in an intermediate conformation, as shown by the position of the viral DNA (yellow). **C.** IN/LEDGF/vDNA/tDNA structure in the strand transfer conformation after release of INI1 and binding to the target DNA (red).

The overall domain organization of the IN tetramer in complex with DNA, LEDGF and INI1-IBD is similar to of the one found in the absence of INI1 [17] except for conformational changes in the N- and C-terminal parts of IN due to their interactions with INI1-IBD. These interactions stabilize an IN conformation that is not compatible with the 3′ processing and integration reaction. In particular, the reorientation of the N- and C-terminal parts of IN induces a rotation of about 40° of the viral DNA as compared to the previously studied 3′ processing complex (17) (**Fig. 4**). The DNA orientation in the IN/LEDGF/INI1-IBD/vDNA complex is thus intermediate to those in the 3′ processing and integration complexes (**Fig. 7**).

These structures are fully consistent with the 3′ processing and concerted integration assays and help to understand the effect of LEDGF and INI1-IBD on the enzymatic activities of IN. In agreement with the structure showing a catalytic inactive conformation, our activity experiments show that the 3′ processing reaction is strongly inhibited by the binding of INI1-IBD to the IN/LEDGF complex. Comparison of the structures of the IN/LEDGF/DNA and IN/LEDGF/INI1-IBD/vDNA complexes shows that the conformational changes necessary to bring the 3′ end of the viral U5 DNA to the IN active site are prevented by INI1-IBD, which sits in the target DNA (tDNA) binding cleft and locks the IN tetramer in a stable constrained conformation. These data provide a structural basis for a new inhibition strategy that could be used in human therapy. These observations also strongly support the ability of IN to adapt its structure, in order to carry out specific functions directed by the partner protein. *In vitro* integration assays showed that the activity of the IN/LEDGF complex is strongly enhanced compared to IN alone, especially in low protein concentrations such as found *in vivo*. Furthermore the sequencing of the full-site integration products showed that the proportion of correctly integrated species with the characteristic 5 bp stagger is higher in the presence of LEDGF. These observations further strengthen the role of LEDGF as a molecular chaperone that organizes the IN tetramer in a functional and highly reactive species. Integration assays were also realized in the presence of INI1-IBD by providing 3′ pre-processed vDNA duplexes to overcome the inhibition of the 3′processing reaction experienced in the presence of INI1-IBD. The presence of INI1-IBD leads to reduced integration events and to a higher integration specificity since unwanted by-products such as linear full site integration or donor/donor integration are strongly reduced. These effects can be clearly explained by the IN/LEDGF/INI1-IBD/vDNA structure, which shows that INI1-IBD sits in the target DNA (tDNA) binding site, competing with the binding of tDNA and leading to reduced integration events as well as inhibition of nonspecific integration.

To establish structure – function relationships we hypothesize that the main structural and functional effects of INI1 on IN is mediated through the INI1 integrase binding domain, which has been shown to be the minimal sequence for the interaction of INI1 with IN [31]. INI1 has been shown to both increase [38], [41], [42] and inhibit [43] viral replication. The two contradictory functions of INI1, inhibition and activation probably occur at different times during the infection cycle. Little is known about the timing of the interaction of cellular proteins with IN. Assuming that INI1-IBD interacts with IN in the same way as the full length protein, the observation that a stable ternary complex between IN, LEDGF and INI1-IBD can be formed suggests that the two cellular proteins may interact with the PIC during the same temporal window. The interaction of INI1 with the PIC is probably an early event since it was shown that INI1 is incorporated in mature virions [39], that HIV-1 infection triggers the nuclear export of INI1 which associates with the incoming HIV-1 PICs [56] and that INI1 is present in the reverse transcription complex [57]. The fact that INI1 expression in a cell line deleted for the gene encoding INI1 increases viral replication in a dose-dependent manner [38] suggests that IN interacts with these newly produced INI1 molecules. Taken together, these observations suggest that the interaction between INI1-IBD and IN we observe in our structure is likely to occur between reverse transcription and 3′ processing and before nuclear translocation. After nuclear internalization, both INI1 and LEDGF are likely to stabilize the highly flexible IN. LEDGF probably stabilizes the IN tetramer while INI1 might prevent non-specific protein interactions and auto-integration on the way to nucleosomes. Moreover, INI1, as part of the SWI/SNF chromatin remodeling complex, is believed to play a role in the control of viral integration through the chromatin reorganization of the host genome [41]. Indeed, *in vitro* experiments showed that stable nucleosomes reconstituted on strongly positioning DNA sequences inhibit the integration of viral DNA and that purified SWI/SNF complexes restore integration, suggesting a coupling between nucleosome remodeling and efficient HIV-1 integration [41]. Thus, SWI/SNF is thought to promote integration in target nucleosomes through its unwinding activity, by producing a suitable nucleosomal DNA for the strand transfer reaction. We speculate that INI1 may be released from IN during the nucleosome remodeling process in order to activate its integration function. In contrast, after INI1 release, LEDGF is likely to remain attached to IN in order to maintain its tetramer organization and to enhance the efficiency of integration (**Fig. 7**).

In the cellular context, it has been shown that the IN-INI1 and IN-LEDGF interactions are advantageous for viral infection. The INI1 and LEDGF cellular proteins would have two major functions in the early state of HIV-1 replication. One function would be to target the PIC to chromatin and nucleosomes through the PWWP domain of LEDGF and to perform nucleosome remodeling through INI1, a part of the SWI/SNF complex. Their second function would be a chaperon function. INI1 would stabilize the PIC in the host cell, by transiently inhibiting the IN enzymatic functions (3′ processing and strand transfer) via capping of the flexible N- and C-terminal parts of the IN tetramer, and thus maintaining IN in a stable constrained conformation on the route to nucleosomes. LEDGF would organize and stabilize an active IN tetramer suitable for specific vDNA integration. Finally, the structure presented in this work, provides the basis for the development of a new type of inhibitor (conformational inhibitor), which is able to block the structural changes of IN required to perform its functions.

Supplementary data are available online: Figures S1–S11 and Methods S1.

## Materials and Methods

### Production and Purification of HIV-1 IN, LEDGF and INI1 (174–289) (INI1-IBD)

The IN/LEDGF complex was produced and purified as previously described [17]. HIS-tagged INI1 (174–289) was cloned in pET expression plasmid and transformed in *Escherichia coli* BL21(DE3) host strain (Novagen) containing pRARE plasmids isolated from Rosetta DE3 strain (Novagen). After the INI1-IBD purification described in **methods S1**, the IN/LEDGF buffer was raised up to 2 M NaCl and 20 mM CHAPS. IN/LEDGF and INI1-IBD were mixed at a 1∶2 molar ratio, respectively, and dialyzed against buffer B [50 mM HEPES pH 7.0, 500 mM NaCl, 5 mM MgCl_2_, 2 mM β-mercaptoethanol]. The ternary complex was concentrated using an Amicon Ultra-15 50 kDa device (Millipore) and loaded at 1 mL/min onto a Superdex 200 HR gel filtration column (GE Healthcare) pre-equilibrated in buffer B. Peak fractions were used directly for electron microscopy and functional tests. Protein concentration was determined using the Bradford colorimetric assay (Bio-Rad). The purity of the complex was checked on SDS-PAGE and DNA contamination by UV spectrum.

### High-Mass MALDI ToF Mass Spectrometry Analysis

High-Mass MALDI mass spectra were obtained using a MALDI-TOF equipped with HM2 TUVO High-Mass retrofit system (CovalX AG, Zürich, Switzerland). The High-Mass retrofit system allows a sensitive detection (sub-µM) of macromolecules up to 1500 kDa with low saturation. The instrument was operated in the linear mode by applying an accelerating voltage of 20 kV and a gain voltage set to 2.95 kV. Mass spectra were acquired by averaging 300 shots (3 different positions into each spot and 100 shots per position). All subsequent mass spectra acquisitions were performed by applying the same laser fluency before and after cross-linking. Further information is provided in **methods S1**.

### Fluorescence Correlation Spectroscopy

Fluorescence correlation spectroscopy (FCS) measurements were performed with in-house setup [58], [59], consisting of an Olympus IX 71 microscope associated with a two-photon excitation at 800 nm, provided by a mode-locked Ti:Sapphire laser (Tsunami, Spectra Physics). Emitted photons were detected with an Avalanche Photodiode (APD SPCM-AQR-14-FC, PerkinElmer Optoelectronics). The normalized autocorrelation function *G*(τ) was calculated on line by a hardware correlator (ALV 5000, ALV GmbH). Multiple FCS runs [60] of short duration (5 s) were performed on solutions of viral DNA tagged with Texas Red (vDNA-TXR), without and with IN/LEDGF. The excitation power was about 5 mW at the sample, in order to provide optimal signal/noise ratio and minimal probe photobleaching [61]. The details of data processing are described in **methods S1**.

### Determination of Binding Constants by Fluorescence Anisotropy

Titration curves were performed in 384 well-plates, containing 20 µL of reaction mixtures composed of (i) 2 nM of a 40 bp double strand DNA with the sequence of the U5 end of HIV-1 DNA and 5′ modified by 6-fluorescein and (ii) increasing concentrations of IN/LEDGF or IN/LEDGF/INI1-IBD. Competition experiments were done using non-modified U5 DNA and non-modified 49 bp double strand DNA with a random sequence. The final buffer contained 150 mM NaCl, 50 mM HEPES pH 7.5, 5 mM MgCl_2_, 1 mM β-mercaptoethanol. After homogenization, the fluorescence anisotropy measurements were performed for 3 minutes in triplicate on a PHERAstar^Plus^ (BMGLab) microplate reader at 20°C, using an excitation polarized wavelength of 470 nm and an emission wavelength of 520 nm. Further information and the calculation of the dissociation constant are provided in **methods S1.**

### IN-LEDGF and IN-LEDGF-INI1-IBD 3′ Processing Activity Monitored by Fluorescence Anisotropy

The reaction was done in a 96 well-plate. One well contained 100µL of reaction mix composed of 10 mM NaCl, 25 mM BisTris pH 6.5, 10 mM MgCl_2_, 5 mM DTT, 50 nM DNA and 200 nM of protein complex. The DNA is a 40 base pair double strand DNA, mimicking the U5 end of HIV-1 DNA and 3′ modified by 6-fluorescein. After homogenization, 50µL of paraffin oil was added on the top of the well to avoid evaporation. Fluorescence anisotropy measurements were performed on a PHERAstar^Plus^ (BMGLab) spectrophotofluorimeter with an excitation polarized wavelength of 470 nm. The reaction was monitored for 6 hours at 37°C. Further information is provided in **methods S1**.

### In vitro Concerted Integration Assay

Standard concerted integration reactions were performed as described previously [41], [62], with some modifications. Briefly, purified HIV-1 IN (0.1 to 12.5 µM in IN monomer), IN/LEDGF complex (0.1 to 12.5 µM in IN monomer) or IN/LEDGF/INI1-IBD complex (12.5 µM in IN monomer) was pre-incubated with both a 246 bp 5′-end-labeled donor DNA (15 ng), containing the processed U3 and U5 LTR sequences and a SupF gene, and the target DNA plasmid pBSK^+^ (150 ng) at 0°C for 20 min in a total volume of 5 µl. Then, the reaction mixture (20 mM HEPES, pH 7.5; 10 mM DTT; 10 mM MgCl_2_; 15% DMSO; 8% PEG, 30 mM NaCl) was added and the reaction proceeded for 120 min at 37°C in a total volume of 10 µL. Incubation was stopped by adding a phenol/isoamyl alcohol/chloroform mix (24/1/25 v/v/v). The aqueous phase was loaded on a vertical 1% agarose gel in the presence of 1% bromophenol blue and 1 mM EDTA. After separation of the products, the gel was treated with 5% TCA for 20 min, dried and autoradiographed. All IN activities were quantified by scanning of the bands (half site plus full site integration products) after gel electrophoresis and autoradiography using the Image J software. Both target DNA and donor plasmids were kind gifts from Dr. K Moreau (Université Claude Bernard-Lyon I, France). The target corresponds to the pBSK^+^ plasmid (Stratagene, La Jolla, California), carrying the zeocin resistance- encoding gene.

The Full Site Integration (FSI) reaction was additionally quantified by cloning the integration products into bacteria using the same protocol as described previously [62]. Briefly, after concerted integration, the products were purified on a DNA purification system column (Promega), as described by the supplier and then introduced into a MC1060/P3 *E. coli* strain which contained ampicillin-, tetracycline- and kanamycin-resistance genes. Both ampicillin- and tetracycline-resistance genes carry an *amb* mutation. These proteins are thus expressed only in the presence of *supF* gene products. Integration clones carrying the *supF* gene were therefore selected in the presence of 40 µg/ml ampicillin, 10 µg/ml tetracycline and 15 µg/ml kanamycin. The integration loci structure was determined by isolating plasmids from quadruple-resistant colonies and PCR sequencing (ABI Prism big dye terminator cycle sequencing ready reaction kit, Applied Biosystems) using the U3 primer (5′-TATGGAAGGGCTAATTCACT-3′) and the U5 primer (5′-TATGCTAGAGATTTTCCACA-3′).

### Electron Microscopy and Image Processing

For negatively stained samples, the purified IN/LEDGF/INI1-IBD complexes were diluted to a concentration of 20 µg/mL in buffer B and crosslinked with 0.1% glutaraldehyde for 5 sec. 10 µL of this preparation were placed on a 10 nm thick carbon film treated by a glow discharge in air. After two minutes of adsorption, the specimen was negatively stained with a 2% (w/v) uranyl acetate solution. Images were recorded under low-dose conditions on a transmission electron microscope (TEM, Philips CM120), equipped with a LaB_6_ cathode and operating at 100 kV at 45,000 X magnification on a Pelletier cooled slow scan CCD camera (Model 794, Gatan, Pleasanton), which resulted in a pixel spacing of 0.37 nm on the object.

For frozen hydrated samples, the complexes were diluted and adsorbed as described above, but were vitrified using an automated plunger equipped with a temperature and humidity controlled chamber (Vitrobot FEI). Images were recorded under low-dose conditions (15–20 e^−/^Å^2^) on a cryo-TEM equipped with a field emission gun operating at 200 kV (Tecnai F20, FEI) and with a side-entry cold stage working at a temperature of −172°C. The image data set was acquired on photographic plates (SO163, Eastman-Kodak) at 50,000 X magnification and at defocus values ranging from 2.7 to 3.9 µm. The micrographs were digitized using a drum scanner (Primescan D7100, Heidelberg) to obtain a final pixel spacing of 0.2 nm. Examples of the initial images are shown in **figure S10**. Class average images obtained after reference-free classification and the corresponding re-projections of the final 3-D model fitting are shown in **figure S11**. Image analysis is described in **methods S1**.

### Model Building and Fitting

Flexible fitting of the atomic structures in the EM maps was done using normal mode analysis [63], [64]. Normal mode flexible and rigid body fitting were performed with the procedure described in **methods S1**, using NORMA [63] and URO [65]. Regularization of the structure was done with REFMAC [66]. Difference maps were calculated using CCP4 [67] and COOT [68] and map superposition was performed with UCSF chimera [69]. Figures were produced with UCSF chimera and Pymol [70]. Further information is provided in **methods S1**.

## Supporting Information

Figure S1 **Superimposition of IN catalytic core domain structures. A.** Superimposition of the structures of the IN catalytic core domain in the Maedi visna virus (MVV) [71] in pink, the Prototype foamy virus (PVF) [72] in cyan, the Human immunodeficiency virus type 2 (HIV-2) [73] in yellow and the Human immunodeficiency virus type 1 (HIV-1) [12] in red. The N-terminal domains are circled in blue. **B**. Superimposition of the structures of the IN catalytic core domain of the Rous sarcoma virus (RSV) [74] in gold, HIV-1 [11] in red and PVF [72] in cyan. The C-terminal domains are circled in red.
(TIF)

Figure S2 **EM structure of PFV integrase.** (**A, B, C**) Three perpendicular surface representation of the EM 3-D envelope of the PFV integrase. (**D**) Fitting of the PFV X-ray structure in the EM map. (**E**) Resulting structure after normal mode flexible fitting. The details of the structure solving are described in supplemental protocols. (**F**) EM Structures of the IN/LEDGF complex in grey and the PFV IN tetramer in gold.
(TIF)

Figure S3 **Comparison of the HIV-1 and PFV intasome.** **A**. Superimposition of the structures of the IN catalytic domain in the HIV-1 IN-LEDGF-DNA complex (blue) [17] and the PFV IN-DNA complex (gold) [16], showing that the viral DNA is collinear in the two complexes. **B**. Same superimposition showing that the target DNA comes from opposite sides in PFV and HIV-1. **C**. Topology of the organization of IN monomers in gold-yellow for PFV and blue for HIV-1. **D**. Schema of the tetramer organization in PFV and HIV1.
(TIF)

Figure S4 **Domain organization of INI1.** Domain organization of INI1 and position on the sequence of the 174–289 fragment used in this study.
(TIF)

Figure S5 **Purification and characterization of the IN/LEDGF/INI1-IBD complex.** Purification and characterization: **A**. IN, LEDGF and INI1-IBD are purified separately. IN and LEDGF are mixed together and the complex is formed by removing the solubilizing agents by dialysis. The complex is then purified on a GST affinity column. The GST tag is cut by the P3C protease and removed on a GST affinity column. The salt concentration is then increased and the IN/LEDGF complex is mixed with INI1-IBD. The CHAPS and salt are then decreased by dialysis. Finally, the IN/LEDGF/INI1-IBD complex is purified on a gel filtration column. The Coomassie blue stained SDS-PAGE demonstrates the homogeneity of the IN/LEDGF/INI1-IBD complex. **B**. IN/LEDGF/INI1-IBD complex analysis by High-Mass MALDI mass spectrometry. **C**. The same analysis after reaction with cross-linking agents. A major peak of (IN)_4_-(LEDGF)_2_-(INI1-IBD)_2_ was observed.
(TIF)

Figure S6 **Determination by two-photon FCS of the binding stoichiometry of U5 vDNA-TXR duplexes to IN/LEDGF.** Determination by two-photon FCS of the binding stoichiometry of U5 vDNA-TXR duplexes to IN/LEDGF. **A**: Autocorrelation curves of U5 vDNA-TXR (40 nM) in the absence (black) and the presence (red) of 26 nM IN/LEDGF are shown, together with their fits (*solid line)* using eq. 1 and 2 in **methods S1** respectively. **B**: Brightness distribution for U5 vDNA-TXR (40 nM) in the absence (black bar) and in the presence of IN/LEGDF (red bar). The histogram was obtained by sorting the measured brightness values (*n*≈60) into different classes of 0.2 kHz in width. The median value is about 0.77 kHz for the free U5 vDNA-TXR duplexes and about 1.5 kHz for the U5 vDNA-TXR/IN/LEDGF complexes. **C:** Normalization of the autocorrelation curves of figure 2A.
(TIF)

Figure S7 **Gel based 3′ processing assay and quantification.** **A:** Determination of the 3′ processing activity for the IN/LEDGF and IN/LEDGF/INI1-IBD complex. The reaction was done using a 5′ 6FAM modified U5 dsDNA of 40 base pairs. For the IN/LEDGF complex we clearly see the vanishing of the 40 mer DNA and the apparition of the band corresponding to the 38 mer DNA, whereas in the presence of INI1 there is no difference with the control (DNA alone), confirming the inhibitory effect of INI1-IBD. The reaction mixture contained 200 µL of reaction mix composed of 23 mM NaCl, 25 mM BisTris pH 6.5, 10 mM MgCl_2_, 5 mM DTT, 125 nM DNA and 500 nM of protein complex. The reaction was stopped at 0, 60, 240 and 360 mn by the addition of 200 µL of STOP buffer (25 mM Tris/HCl pH 7.5, 25 mM EDTA, 0,125 mg/ml Glycogen, 800 mM NaOAc). The DNA was then precipitated by adding 1 ml of EtOH and kept at −20° overnight. After centrifugation, the DNA pellets were resuspended in 40 µL of loading dye (95% formamide; 20 mM EDTA; 0.1% SDS; 0.025% Bromophenol Blue; 0.025% Xylene Cyanol). The samples wereloaded on a 20% polyacrylamide, 7 M urea denaturing DNA gel and the migration was performed overnight at 500 V. The gel was then scanned on a Typhoon 8600 imager (Molecular Dynamics). **B:** Quantification of the bands on the gel in S7A. Band intensities were quantified using the ImageJ software. For each lane, the relative proportion of processed and non-processed DNA was calculated and represented as a percentage of the total lane intensity. It clearly shows the inhibition of the 3′ processing in the presence of INI1-IBD.
(TIF)

Figure S8 **Quantification of the integration assay shown in** **Fig. 2D** **.** **A:** Intensity of the load band on the autoradiography of the gel shown in figure 2d. **B:** Intensity of the d/d (green) linear FSI (red) and HIS+FSI (blue) bands on the autoradiography of the gel shown in figure 2d. In the presence of INI1-IBD, a reduction of the integration events was observed as well as an inhibition of by-product formation such as d/d or linear FSI molecules.
(TIF)

Figure S9 **Resolution determination.** Fourier Shell Correlation Function obtained by comparing two distinct IN/LEDGF/INI1-IBD/DNA reconstructions obtained by splitting the data set in two. The 0.5 FSC criterion gives a resolution of 24 Å, whereas the Rosenthal and Henderson criterion (0.145 FSC) gives a resolution of 18 Å.
(TIF)

Figure S10 **Electron micrograph of unstained complexes.** Electron micrograph of unstained complexes recorded at low temperature on a cryo electron microscope operating at 200 kV. The particles are circled in the right panel.
(TIF)

Figure S11 **Class averages and re-projections of the final 3-D model. Rows 1, 3, 5:** Gallery of frozen hydrated class averages obtained after reference-free classification and three cycles of alignment/classification that used the best class averages of the previous classification as new alignment references. **Rows 2, 4, 6:** Re-projections of the final 3-D model with the best fit to the initial class averages.
(TIF)

Methods S1 **Detailed information on the experimental methods used (Production and Purification, High-Mass MALDI ToF, Fluorescence Correlation Spectroscopy, Fluorescence Anisotropy, Cryo-electron Microscopy) is provided.**
(PDF)
