# Supplementary figures and images for: Structural and Functional Role of INI1 and LEDGF in the HIV-1 Preintegration Complex
Source: PLoS One. 2013 Apr 11;8(4):e60734. doi: 10.1371/journal.pone.0060734 (PMC3623958; doi:10.1371/journal.pone.0060734)

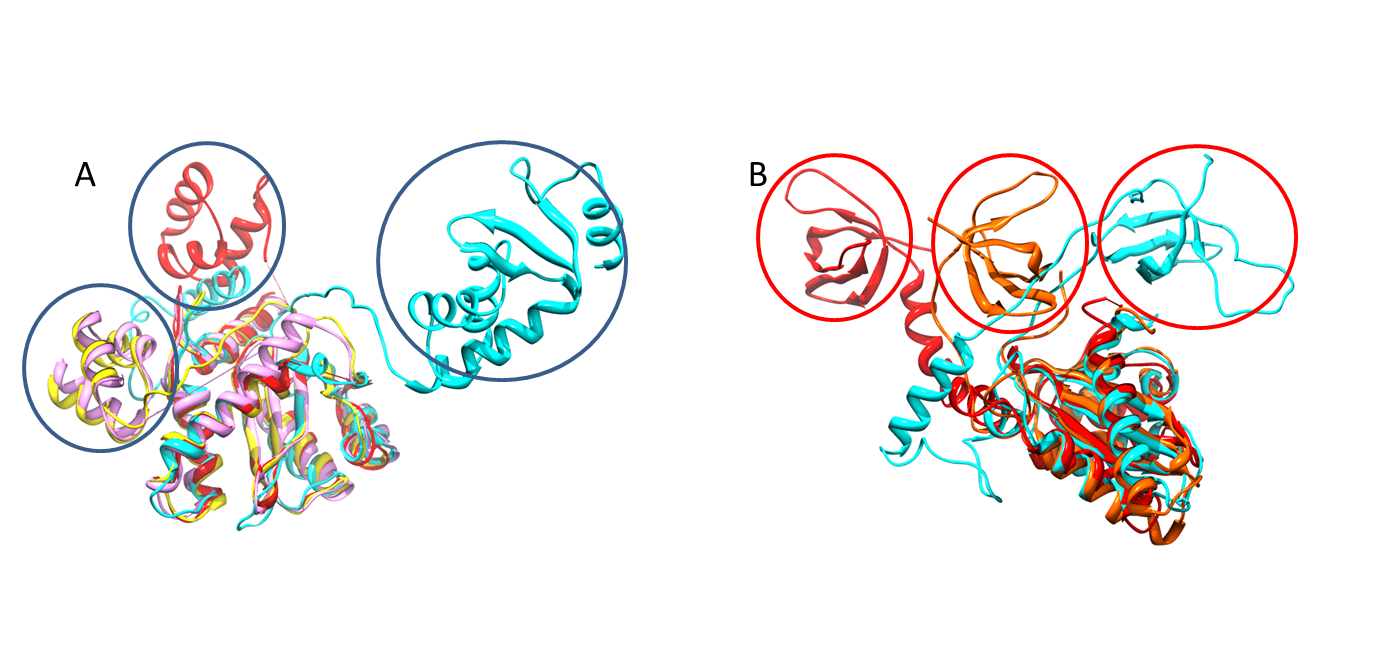

Supplement: Figure S1 — Superimposition of IN catalytic core domain structures. A. Superimposition of the structures of the IN catalytic core domain in the Maedi visna virus (MVV) [71] in pink, the Prototype foamy virus (PVF) [72] in cyan, the Human immunodeficiency virus type 2 (HIV-2) [73] in yellow and the Human immunodeficiency virus type 1 (HIV-1) [12] in red. The N-terminal domains are circled in blue. B. Superimposition of the structures of the IN catalytic core domain of the Rous sarcoma virus (RSV) [74] in gold, HIV-1 [11] in red and PVF [72] in cyan. The C-terminal domains are circled in red. (TIF) [file pone.0060734.s001.tif]

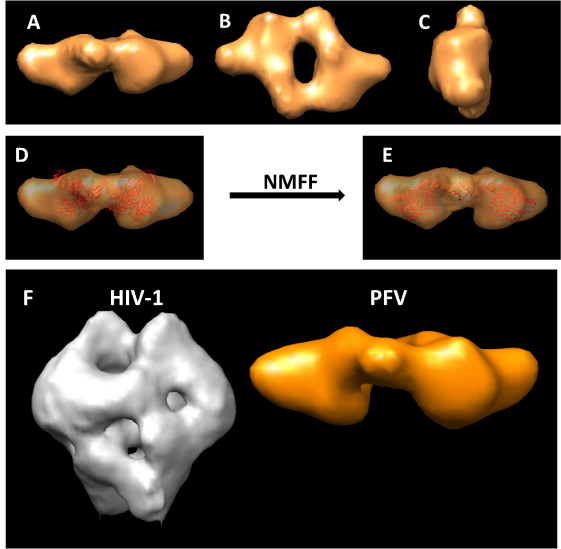

Supplement: Figure S2 — EM structure of PFV integrase. (A, B, C) Three perpendicular surface representation of the EM 3-D envelope of the PFV integrase. (D) Fitting of the PFV X-ray structure in the EM map. (E) Resulting structure after normal mode flexible fitting. The details of the structure solving are described in supplemental protocols. (F) EM Structures of the IN/LEDGF complex in grey and the PFV IN tetramer in gold. (TIF) [file pone.0060734.s002.tif]

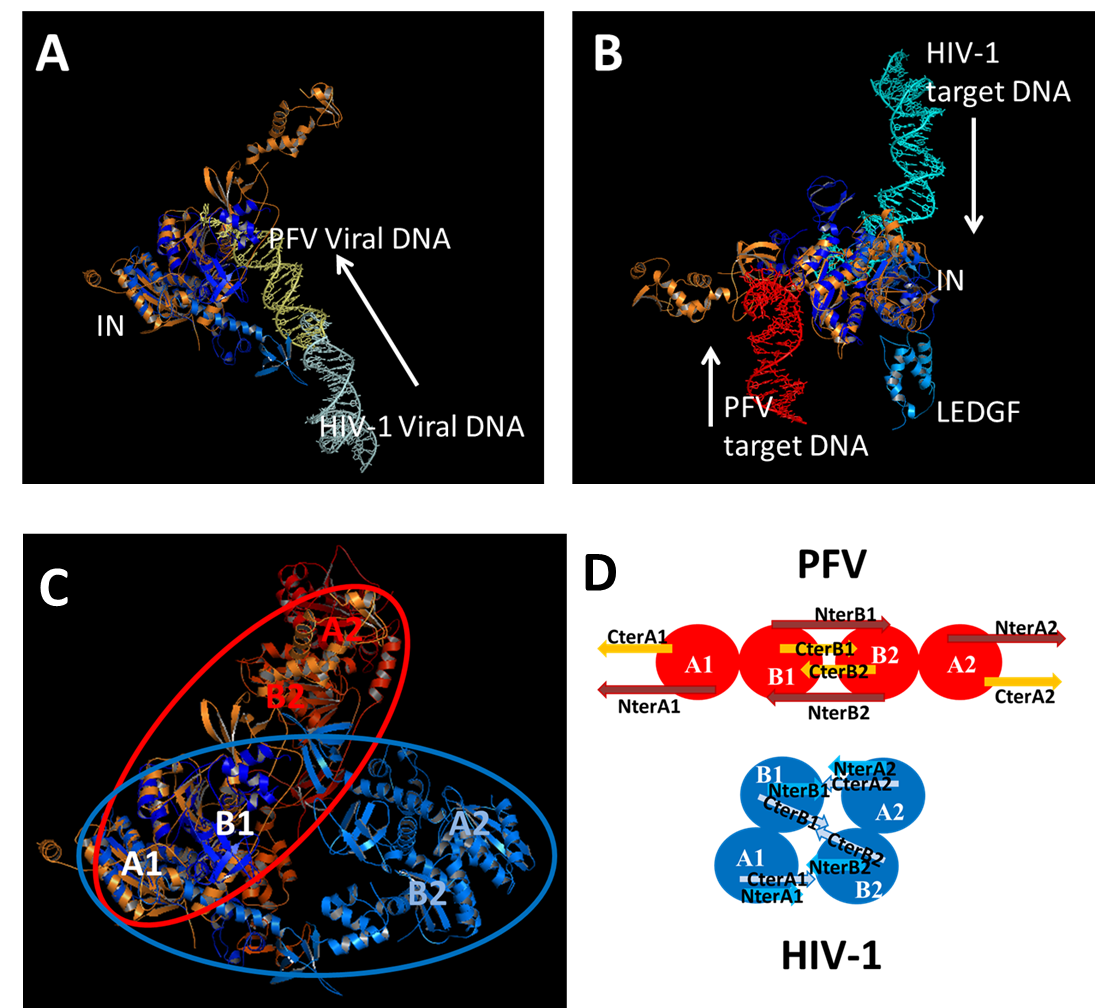

Supplement: Figure S3 — Comparison of the HIV-1 and PFV intasome. A. Superimposition of the structures of the IN catalytic domain in the HIV-1 IN-LEDGF-DNA complex (blue) [17] and the PFV IN-DNA complex (gold) [16], showing that the viral DNA is collinear in the two complexes. B. Same superimposition showing that the target DNA comes from opposite sides in PFV and HIV-1. C. Topology of the organization of IN monomers in gold-yellow for PFV and blue for HIV-1. D. Schema of the tetramer organization in PFV and HIV1. (TIF) [file pone.0060734.s003.tif]

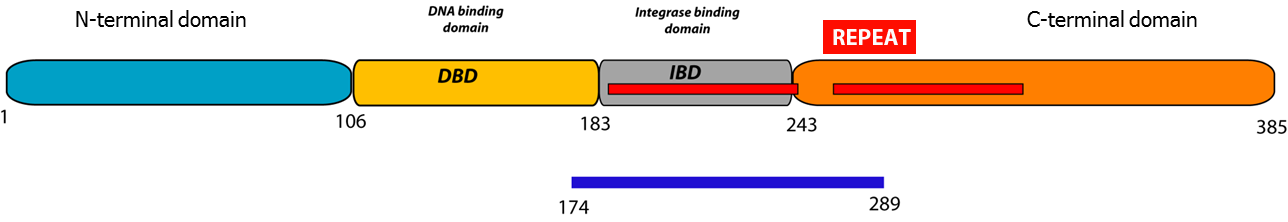

Supplement: Figure S4 — Domain organization of INI1. Domain organization of INI1 and position on the sequence of the 174–289 fragment used in this study. (TIF) [file pone.0060734.s004.tif]

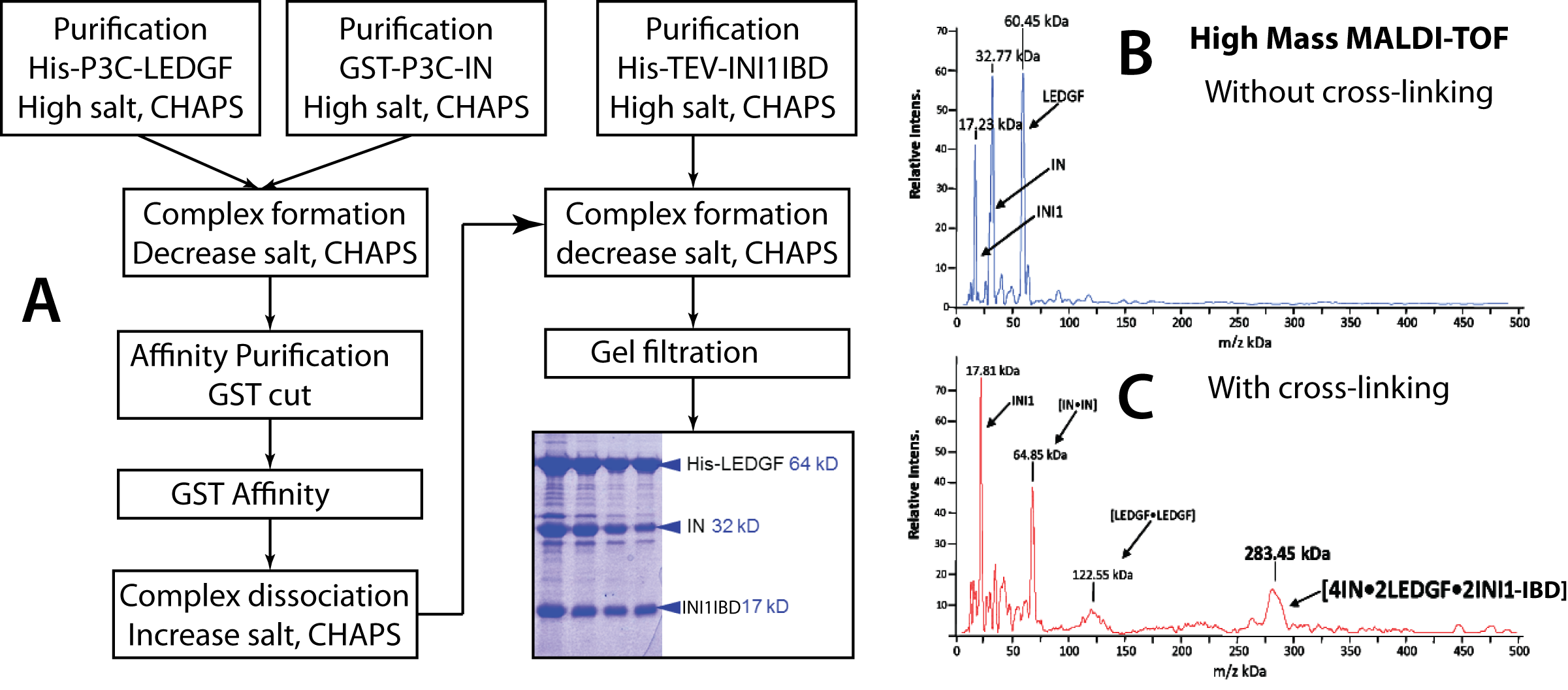

Supplement: Figure S5 — Purification and characterization of the IN/LEDGF/INI1-IBD complex. Purification and characterization: A. IN, LEDGF and INI1-IBD are purified separately. IN and LEDGF are mixed together and the complex is formed by removing the solubilizing agents by dialysis. The complex is then purified on a GST affinity column. The GST tag is cut by the P3C protease and removed on a GST affinity column. The salt concentration is then increased and the IN/LEDGF complex is mixed with INI1-IBD. The CHAPS and salt are then decreased by dialysis. Finally, the IN/LEDGF/INI1-IBD complex is purified on a gel filtration column. The Coomassie blue stained SDS-PAGE demonstrates the homogeneity of the IN/LEDGF/INI1-IBD complex. B. IN/LEDGF/INI1-IBD complex analysis by High-Mass MALDI mass spectrometry. C. The same analysis after reaction with cross-linking agents. A major peak of (IN)4-(LEDGF)2-(INI1-IBD)2 was observed. (TIF) [file pone.0060734.s005.tif]

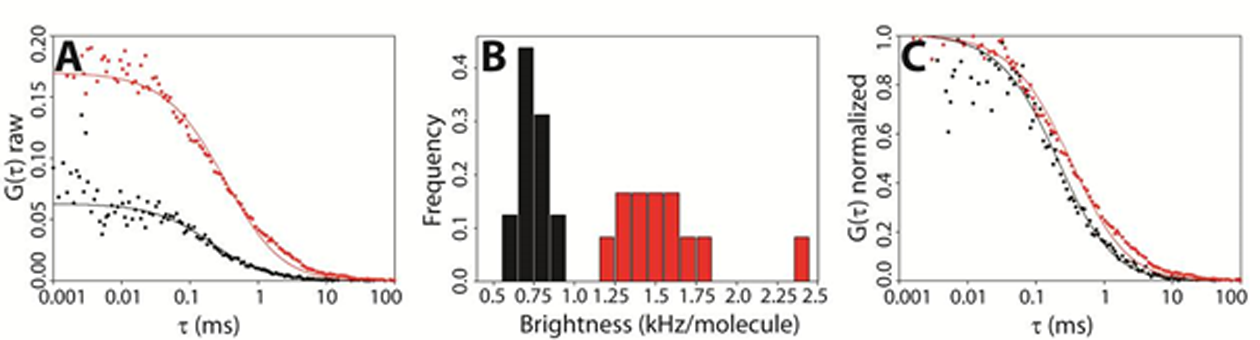

Supplement: Figure S6 — Determination by two-photon FCS of the binding stoichiometry of U5 vDNA-TXR duplexes to IN/LEDGF. Determination by two-photon FCS of the binding stoichiometry of U5 vDNA-TXR duplexes to IN/LEDGF. A: Autocorrelation curves of U5 vDNA-TXR (40 nM) in the absence (black) and the presence (red) of 26 nM IN/LEDGF are shown, together with their fits (solid line) using eq. 1 and 2 in methods S1 respectively. B: Brightness distribution for U5 vDNA-TXR (40 nM) in the absence (black bar) and in the presence of IN/LEGDF (red bar). The histogram was obtained by sorting the measured brightness values (n≈60) into different classes of 0.2 kHz in width. The median value is about 0.77 kHz for the free U5 vDNA-TXR duplexes and about 1.5 kHz for the U5 vDNA-TXR/IN/LEDGF complexes. C: Normalization of the autocorrelation curves of figure 2A. (TIF) [file pone.0060734.s006.tif]

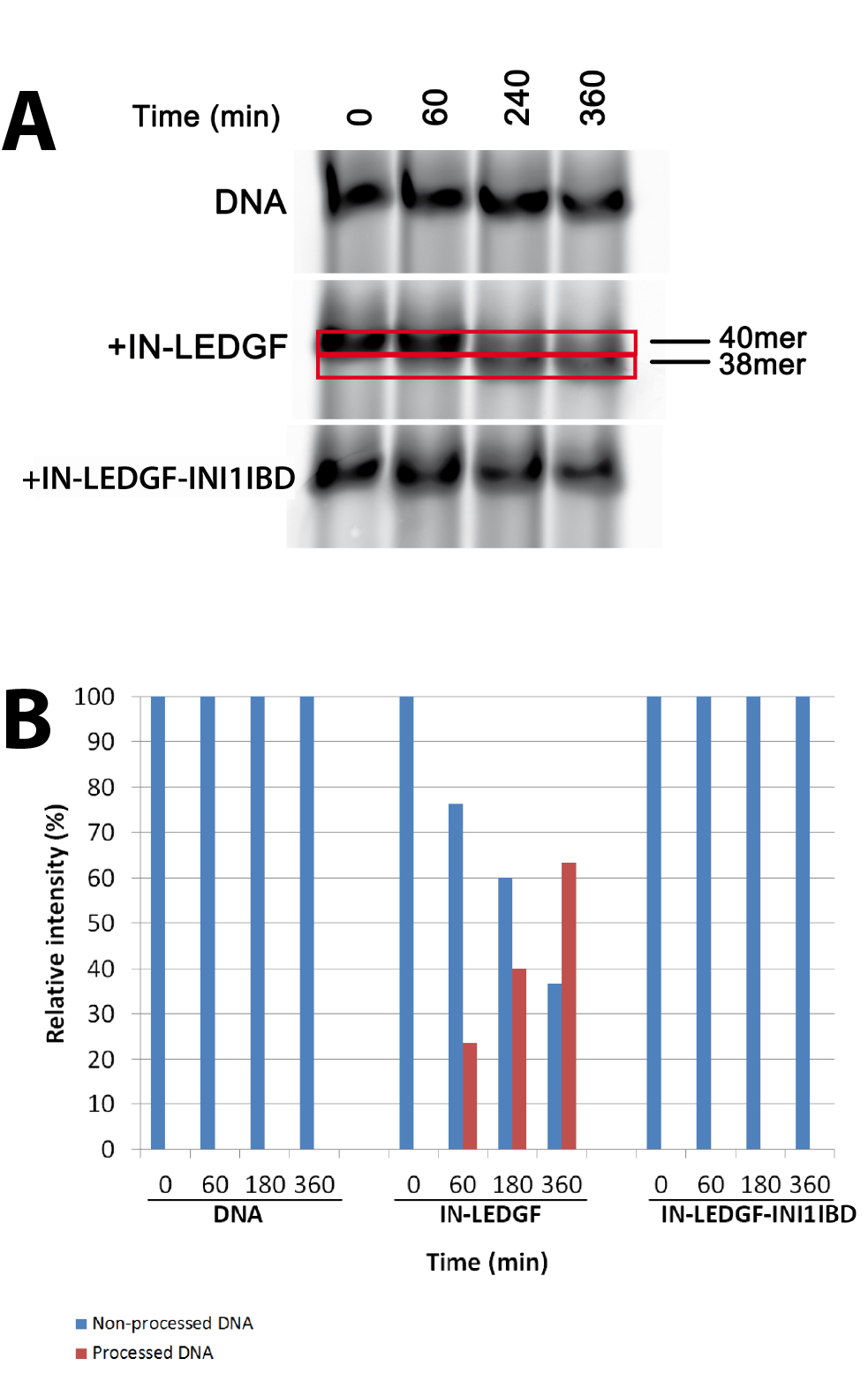

Supplement: Figure S7 — Gel based 3′ processing assay and quantification. A: Determination of the 3′ processing activity for the IN/LEDGF and IN/LEDGF/INI1-IBD complex. The reaction was done using a 5′ 6FAM modified U5 dsDNA of 40 base pairs. For the IN/LEDGF complex we clearly see the vanishing of the 40 mer DNA and the apparition of the band corresponding to the 38 mer DNA, whereas in the presence of INI1 there is no difference with the control (DNA alone), confirming the inhibitory effect of INI1-IBD. The reaction mixture contained 200 µL of reaction mix composed of 23 mM NaCl, 25 mM BisTris pH 6.5, 10 mM MgCl2, 5 mM DTT, 125 nM DNA and 500 nM of protein complex. The reaction was stopped at 0, 60, 240 and 360 mn by the addition of 200 µL of STOP buffer (25 mM Tris/HCl pH 7.5, 25 mM EDTA, 0,125 mg/ml Glycogen, 800 mM NaOAc). The DNA was then precipitated by adding 1 ml of EtOH and kept at −20° overnight. After centrifugation, the DNA pellets were resuspended in 40 µL of loading dye (95% formamide; 20 mM EDTA; 0.1% SDS; 0.025% Bromophenol Blue; 0.025% Xylene Cyanol). The samples wereloaded on a 20% polyacrylamide, 7 M urea denaturing DNA gel and the migration was performed overnight at 500 V. The gel was then scanned on a Typhoon 8600 imager (Molecular Dynamics). B: Quantification of the bands on the gel in S7A. Band intensities were quantified using the ImageJ software. For each lane, the relative proportion of processed and non-processed DNA was calculated and represented as a percentage of the total lane intensity. It clearly shows the inhibition of the 3′ processing in the presence of INI1-IBD. (TIF) [file pone.0060734.s007.tif]

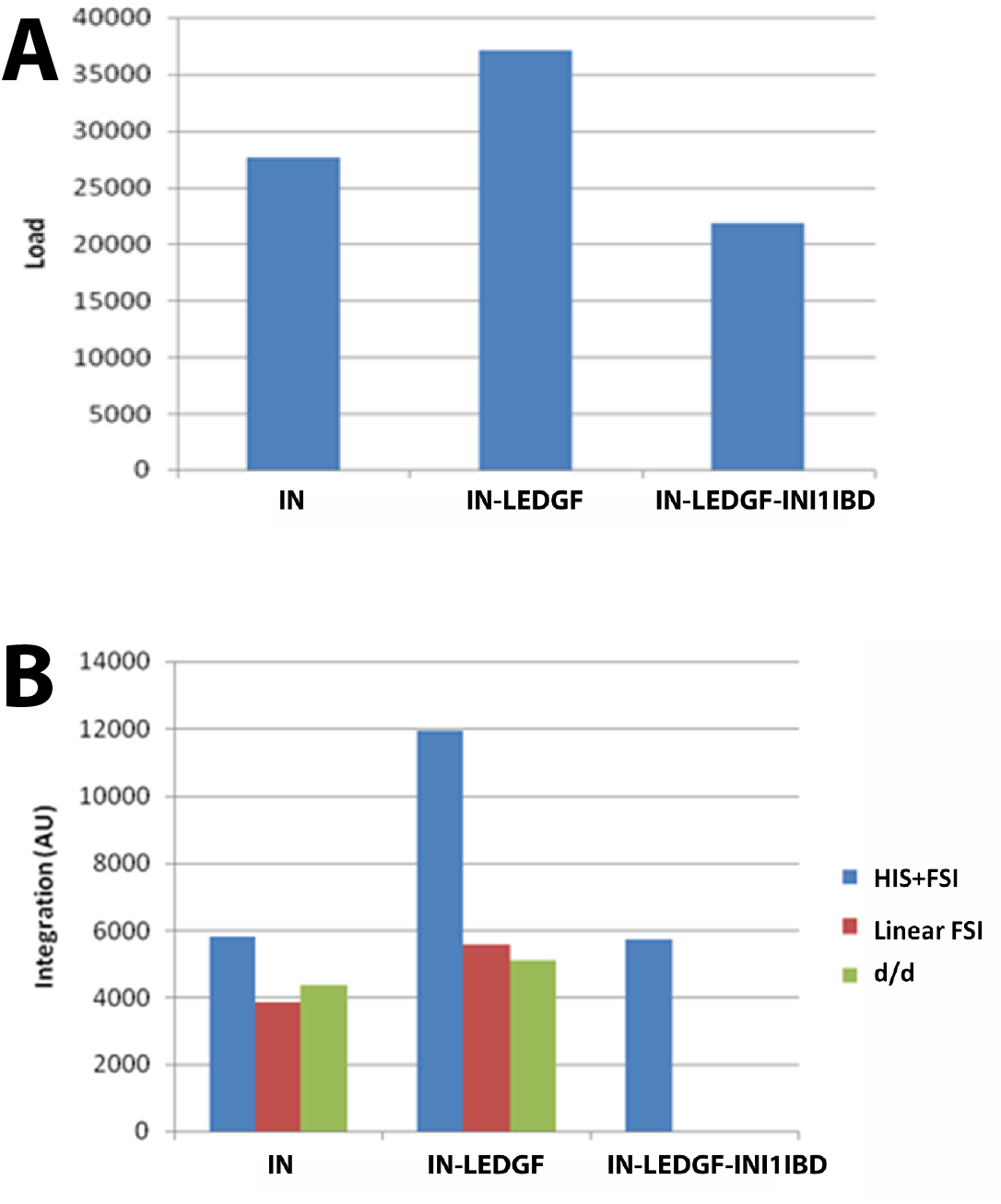

Supplement: Figure S8 — Quantification of the integration assay shown in Fig. 2D . A: Intensity of the load band on the autoradiography of the gel shown in figure 2d. B: Intensity of the d/d (green) linear FSI (red) and HIS+FSI (blue) bands on the autoradiography of the gel shown in figure 2d. In the presence of INI1-IBD, a reduction of the integration events was observed as well as an inhibition of by-product formation such as d/d or linear FSI molecules. (TIF) [file pone.0060734.s008.tif]

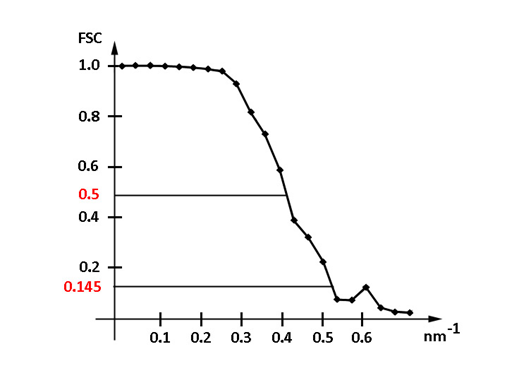

Supplement: Figure S9 — Resolution determination. Fourier Shell Correlation Function obtained by comparing two distinct IN/LEDGF/INI1-IBD/DNA reconstructions obtained by splitting the data set in two. The 0.5 FSC criterion gives a resolution of 24 Å, whereas the Rosenthal and Henderson criterion (0.145 FSC) gives a resolution of 18 Å. (TIF) [file pone.0060734.s009.tif]

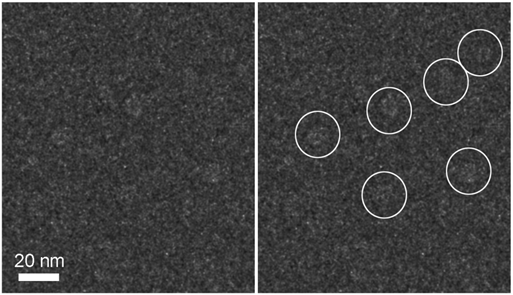

Supplement: Figure S10 — Electron micrograph of unstained complexes. Electron micrograph of unstained complexes recorded at low temperature on a cryo electron microscope operating at 200 kV. The particles are circled in the right panel. (TIF) [file pone.0060734.s010.tif]

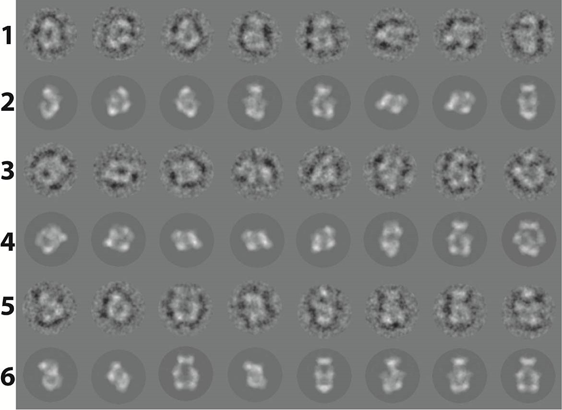

Supplement: Figure S11 — Class averages and re-projections of the final 3-D model. Rows 1, 3, 5: Gallery of frozen hydrated class averages obtained after reference-free classification and three cycles of alignment/classification that used the best class averages of the previous classification as new alignment references. Rows 2, 4, 6: Re-projections of the final 3-D model with the best fit to the initial class averages. (TIF) [file pone.0060734.s011.tif]
