# Supplementary material for: Structural and Functional Role of INI1 and LEDGF in the HIV-1 Preintegration Complex
Source: PLoS One. 2013 Apr 11;8(4):e60734. doi: 10.1371/journal.pone.0060734 (PMC3623958; doi:10.1371/journal.pone.0060734)
Supplement: Methods S1 — Detailed information on the experimental methods used (Production and Purification, High-Mass MALDI ToF, Fluorescence Correlation Spectroscopy, Fluorescence Anisotropy, Cryo-electron Microscopy) is provided. (PDF) [file pone.0060734.s012.pdf]

Structural and functional role of INI1 and LEDGF in the HIV-1 preintegration complex.

Benoit Maillot[1*], Nicolas Lévy[1*], Sylvia Eiler[1], Corinne Crucifix[1], Florence Granger[1], Ludovic Richert[2], Pascal Didier[2], Julien Godet[2], Karine Pradeau-Aubreton[1], Stéphane Emiliani[3], Alexis Nazabal[4], Paul Lesbats[5], Vincent Parissi[5], Yves Mely[2], Dino Moras[1], Patrick Schultz[1] and Marc Ruff[1#].

[1]Institut de Génétique et de Biologie Moléculaire et Cellulaire, Département de Biologie Structurale intégrative, Université de Strasbourg, U596 INSERM, UMR7104 CNRS, Illkirch, France

[2]Laboratoire de Biophotonique et Pharmacologie. UMR 7213 CNRS, UDS, Faculté de Pharmacie, Illkirch, France

[3]Institut Cochin, Université Paris Descartes, CNRS (UMR8104), INSERM (U567), Paris, France

[4]CovalX AG, Zürich-Schlieren, Switzerland

[5]Laboratoire de Microbiologie Fondamentale et Pathogénicité, CNRS (UMR5234), Université de Bordeaux 2, Bordeaux, France

*BM and NL contributed equally to this work

[#]To whom correspondence should be addressed. Tel: 33 3 88 65 33 49; Fax: 33 3 88 65 32 76; Email: ruff@igbmc.fr

Present address:
Benoit Maillot: European Synchrotron Radiation Facility, Structural Biology Group, Grenoble, France; **Paul Lesbats**: Cancer Research UK London Research Institute, Clare Hall Laboratories, South Mimms, Hertfordshire UK.

SUPPLEMENTARY INFORMATION

This file incudes:

Supplementary methods

Supplementary references

Supplementary Methods: 3
Production and purification of INI1 (174-290) 3
High-Mass MALDI ToF: Cross linking reactions protocol 3
Fluorescence correlation spectroscopy 4
Data processing: 4
DNA sequence and hybridization 6
Fluorescence anisotropy spectroscopy: determination of binding constants 7
Data processing: 7
DNA sequence and hybridization 7
Fluorescence anisotropy spectroscopy: 3'processing assay 8
Data processing: 8
DNA hybridization: 8
Cryo-electron microscopy 9
Data processing: 9
U5 viral DNA duplex used for the complex formation 9
Model building and fitting 10
Prototype Foamy Virus EM structure solving 10
Supplementary References: 11

Supplementary Methods:

Production and purification of INI1 (174-290)

Cells transformed with the pET carrying the INI1-IBD gene and pRARE plasmids were grown at 37°C in 1L of LB, containing 10% sucrose, 100µg/mL ampicillin and 17µg/mL chloramphenicol up to an OD_{600} of 0.6. Protein expression was then induced with 0.5mM isopropyl-β-D-thiogalactopyranoside and cells were further grown overnight at 18°C before harvesting. Cells were suspended in 50mL of buffer A [50mM HEPES pH 7.0, 2M NaCl, 5mM $MgCl_2$, 20mM CHAPS, 2mM β-mercaptoethanol, 5% glycerol, 1mM PMSF] per liter of culture and lysed by a Microfluidizer processor (Microfluidics). The lysate was loaded on a 5-mL Hitrap-Ni column (GE Healthcare) and the protein was eluted by a linear gradient of imidazole (from 1mM to 500mM).

High-Mass MALDI ToF: Cross linking reactions protocol

The cross-linking reactions were performed using a solution containing different cross-linkers specific for amino and sulfhydryl groups. The cross linkers used were 1,1'-(suberoyldioxy) bisazabenzotriazole (SBAT, di(3H-[1,2,3]triazolo[4,5-b]pyridin-3-yl) octanedioate), glutaroyldioxy bisazabenzotriazole (GBAT, di(3H-[1,2,3]triazolo[4,5-b]-pyridin-3-yl) glutarate), decanoyldioxy bisazabenzotriazole (DBAT, di(3H-[1,2,3]triazolo[4,5-b]pyridin-3-yl) decanedioate) (K200 MALDI MS analysis Kit, CovalX AG, Zürich, Switzerland). The complex was cross-linked in the 0.6-4 µM range in a volume of 10 µL. After addition of the reagents, the sample was incubated 3h to achieve a complete reaction. The sample containing the cross-linked complex was mixed with a matrix solution (1:2 v/v) of sinapic acid (10mg/mL) containing 50% acetonitrile (v/v) diluted in deionized water with 0.1% TFA. After mixing, 1µL of the mixture was deposited on the MALDI target using the dried-droplet method.

Fluorescence correlation spectroscopy

Data processing:

We recorded the fluorescence intensity fluctuations of the molecular species crossing the small volume provided by a two-photon excitation. FCS determines the diffusion coefficient, the average number, and the individual molecular brightness (product of the molecular extinction coefficient by the fluorescence quantum yield and the detection efficiency of the microscope) of the diffusing fluorescent species in solution [1]. In our case, the brightness analysis was of special interest to determine the number of fluorescent U5 vDNA-TXR duplexes bound to IN/LEDGF, since the binding of two U5 vDNA-TXR duplexes to LEGDF is expected to increase the brightness by a factor of two compared to U5 vDNA-TXR duplexes alone [1].

FCS autocorrelation curves were individually fitted with the standard 3D diffusion model for one (eq 1) or two (eq 2) diffusing species:

$$G(\tau)= \left(\frac{1}{N}\right) \times \left[\frac{1}{\left(1+\frac{\tau}{\tau_D}\right)} \times \frac{1}{\sqrt{\left(1+\frac{\tau}{s^2 \times \tau_D}\right)}}\right] \quad (1)$$

$$G(\tau)= \left(\frac{1}{N_1B_1+N_2B_2}\right)^2 \times \left(\left[N_1B_1^2\frac{1}{\left(1+\frac{\tau}{\tau_{D_1}}\right)} \times \frac{1}{\sqrt{\left(1+\frac{\tau}{s^2 \times \tau_{D_1}}\right)}}\right] + \left[N_2B_2^2\frac{1}{\left(1+\frac{\tau}{\tau_{D_2}}\right)} \times \frac{1}{\sqrt{\left(1+\frac{\tau}{s^2 \times \tau_{D_2}}\right)}}\right]\right) \quad (2)$$

where N, N_1, N_2 are the average number of the fluorescent species diffusing through the excitation volume, τ is the lag time, τ_D, τ_{D1}, τ_{D2}, are the average residence time of the fluorescent species in the focal volume, and s is the ratio of the axial to lateral radii of the excitation volume. The experimental curves were analyzed using the Global-analysis approach [2]. The fitting parameters were obtained with a in house Matlab (Mathworks) function using the Marquardt-Levenberg nonlinear methods of least squares [3] to automatically process the data. The molecular brightness of the fluorescent species diffusing through the excitation volume is defined as the number of photons emitted per second per species for a given excitation intensity. This parameter was obtained by dividing the average

fluorescence intensity $\langle F \rangle$ by the average number (*N*) of fluorescent species in the focal volume for each individual curve (5 s acquisition) or through a fit of the autocorrelation curves to eq. 2. The point spread function (*i.e.* focal volume) of the setup was determined from a *z*-scan on a fluorescein-labeled bead (diameter 20 nm). The measured lateral (0.34 µm) and axial (1.1 µm) resolutions defined an excitation volume of 0.2 fL for an 800 nm excitation wavelength. The system was calibrated with a 50 nM solution of rhodamine B (RhoB). Assuming a diffusion constant $D_{\mathrm{RhoB}} = 2.8 \times 10^{-6}$ $cm^2 \cdot s^{-1}$ for RhoB [4], the diffusion constants D_{DNA} *and* $D_{DNA(B)}$ for respectively, the vDNA alone and the vDNA bound to IN/LEDGF, were calculated using $D_{DNA(B)} = D_{\mathrm{RhoB}} \times \tau_{D,\ \mathrm{RhoB}}/\tau_{D,DNA(B)}$, where $\tau_{D,\ \mathrm{RhoB}}$ and $\tau_{D,DNA(B)}$ are the average diffusion times in the focal volume of RhoB and vDNA-TXR alone or DNA bound to the IN/LEDGF complex. The diffusion coefficient of a molecular species is related to its hydrodynamic radius, R by:

$$R = \frac{kT}{6\pi n D} \quad (3)$$

where k is the Boltzmann constant, T is the temperature and η is the viscosity of the solvent. The radius is related to the molecular weight MW of the molecule with a specific gravity $\bar{v}$ by:

$$R = \sqrt[3]{\left[\frac{3 \times MW \times \bar{v}}{4\pi}\right]} \quad (4)$$

showing that the diffusion coefficient is weakly dependent on the molecular weight. Finally, the ratio $MW_{DNA(B)}/MW_{DNA}$ of the molecular weights between the vDNA-TXR bound to the IN/LEDGF and vDNA-TXR alone is estimated by [2]:

$$\frac{MW_{DNA(B)}}{MW_{DNA}} \cong \left[\frac{D_{DNA(B)}}{D_{DNA}}\right]^3 \quad (5)$$

The calculation of the theoretical diffusion time of the $IN_4/LEGDF_2/vDNA_2$ complex was performed by approximating it as a prolate ellipsoid, with dimensions (15.5 nm × 14 nm × 10 nm) given by electron microscopy. The theoretical diffusion coefficient D_{th} is given by [5]:

$$D = D_0 \frac{\rho^{2/3}}{\sqrt{\rho^2 - 1}} \times \arctan(\sqrt{1-\rho^2}) \qquad (6)$$

where ρ is the ratio between $R_{\parallel}$, the radius of the major axis to $R_{\perp}$, the radius of the minor axis, D_0 is the diffusion coefficient of a sphere of radius R_0 with the same volume as the ellipsoid. D_0 is calculated from equation (3), using a R_0 value given by [5]:

$$R_0{}^3 = R_{\parallel} \times R_{\perp}{}^2. \qquad (7)$$

DNA sequence and hybridization

The two complementary strands were hybridized equimolarly in buffer (50mM NaCl, 10mM BisTris pH 6.5) at 90°C during 10min. The temperature was then decreased in 14 hours down to 4°C.

Specific U5 vDNA 40 pb labeled at its 5' position by TXR

```
[TXR]-5'-GACTACGGTTCAAGTCAGCGTGTGGAAAATCTCTAGCAGT-3'
         3'-CTGATGCCAAGTTCAGTCGCACACCTTTTAGAGATCGTCA-5'
```

Fluorescence anisotropy spectroscopy: determination of binding constants

Data processing:

The Scatchard equation was rewritten to fit the anisotropy data [6], as follows:

$$r = r_0 + \frac{(r_f - r_0)}{n} \times \left\{ \frac{[1+K_{app}(nN_t+L_t)] - \sqrt{[1+K_{app}(nN_t+L_t)]^2 - 4nK_{app}{}^2 N_t L_t}}{2K_{app}N_t} \right\} \quad (8)$$

where L_t and N_t designate the total concentration of IN/LEDGF or IN/LEDGF/INI1-IBD complex and U5 vDNA-FAM, respectively. r_f represents the anisotropy value at the plateau when all the peptide is bound, whereas r_0 and r correspond to the anisotropy values of U5 vDNA-FAM in the absence and in the presence of a given concentration of IN/LEDGF or INLEDGF/INI1-IBD, respectively. K_{app} and n correspond to the apparent equilibrium binding constant and the number of protein binding sites, respectively. The number of binding sites was 0.5, as determined from structural and FCS data. The fitting of the data was done using the Origin® software. The K_d value was calculated as $1/K_{app}$.

DNA sequence and hybridization

The two complementary strands are hybridized equimolarly in buffer (50mM NaCl, 10mM BisTris pH6.5) at 90°C during 10min. The temperature is decreased in 14 hours down to 4°C.

Specific U5 vDNA 40 pb labeled at 5' position by 6FAM

6FAM-5′-GACTACGGTTCAAGTCAGCGTGTGGAAAATCTCTAGCAGT-3′

3′-CTGATGCCAAGTTCAGTCGCACACCTTTTAGAGATCGTCA-5′

Non-specific DNA 49 bp labeled at 3' position by 6FAM

5′-AGTTAAGTGCTGAATTATGATAGTAATCAATATCTACTCCTAACCTCTT-3′-6FAM

3′-TCAATTCACGACTTAATACTATCATTAGTTATAGATGAGGATTGGAGAA-5′

Fluorescence anisotropy spectroscopy: 3'processing assay

Data processing:

The signal was normalized according to the formula R= (r(max)-Δr)/(r(max)-r(GT)) [7], where r(GT) is the anisotropy value of the dinucleotide GT[FAM], r(max) is the anisotropy value of the complex with the inactive mutant integrase (D64E) and Δr is the measured anisotropy.

DNA hybridization:

The two complementary strands are hybridized equimolarly in buffer (50mM NaCl, 10mM BisTris pH 6.5) at 90°C during 10min. The temperature is decreased in 14 hours down to 4°C.

Specific U5 vDNA 40 pb labeled at 3' position by 6FAM

```
5'-GACTACGGTTCAAGTCAGCGTGTGGAAAATCTCTAGCAGT-3'-[6FAM]
3'-CTGATGCCAAGTTCAGTCGCACACCTTTTAGAGATCGTCA-5'
```

Cryo-electron microscopy

Data processing:

Image analysis was performed using the IMAGIC software package [8] (Image Science Software), as described earlier [9]. For the negatively stained dataset, images were aligned against references obtained by reprojecting the previously published IN/LEDGF model [10]. In the case of the cryoEM images, the CTF was corrected for phase inversion using the EMAN software [11]. The dataset was analyzed independently of any previous model and a reference-free clustering strategy was used to obtain the first alignment references. After 6 iterative rounds of alignment/classifications/class averaging, a stable clustering of the images in 800 classes was obtained. Angular assignment of the class averages was performed using sinogram correlation functions [8] and the initial 3-D model obtained by weighted back projection was refined by multiple rounds of alignment using reprojections of the model as references, followed by standard clustering and 3-D reconstruction steps. The position of the two-fold symmetry axis was determined by finding the best cross correlation value between the 3-D model rotated by successive 5° steps and its 180° rotamer. Two-fold symmetry was then imposed and the resulting model was further refined. Resolution was assessed using the Fourier Shell Correlation (FSC) function between two 3-D reconstructions, obtained by splitting the data set in two and by using the 0.145 FSC criterion [12].

U5 viral DNA duplex used for the complex formation

The DNA is a 21 base pair double-stranded viral DNA, mimicking the U5 end of HIV-1 DNA

```
5'-GTGTGGAAAATCTCTAGCAGT-3’
3’-CACACCTTTTAGAGATCGTCA-5’
```

Model building and fitting

The initial model is the structure of the IN/LEDGF/DNA complex, which has been positioned by the superposition of the IN/LEDGF/DNA map with the IN/LEDGF/INI1-IBD/DNA map (CC=0.65). This model (without DNA) was then refined by NMFF using NORMA with 3 normal modes with a map masked at 10 Å around the molecule followed by structure regularization (CC = 76.0). The DNA molecules were then readily fitted into the map, by a rotation of about 40° of the viral DNA from its position in the IN/LEDGF/DNA strand transfer model (**Fig. S9B**) to fill the empty density in the IN/LEDGF/INI1-IBD/DNA difference map. The C-terminal IN domain interacting with DNA was rebuilt in COOT. The full model was then refined by NMFF using NORMA, first using three normal modes in a map masked at 5 Å around the molecule (CC = 76.3), followed by a one mode NMFF in a map masked with a 10 Å wide mask (CC=90.2). The structure was then regularized using CCP4 REFMAC.

Prototype Foamy Virus EM structure solving

The purified PFV integrase was diluted to a concentration of 20 µg/ml in a buffer containing 0.5M NaCl, 50 mM HEPES pH=7.3, 5mM MgCl2 and 2mM BetaMercaptoethanol. 10µL microliters of this preparation were extemporaly cross-linked with a final glutaraldehyde concentration of 0.2% and placed on a 10 nm thick carbon film previously treated by a glow discharge in air. After two minutes of adsorption, the grid was negatively stained with a 2 % (w/v) uranyl acetate solution. Images were recorded at room temperature on a Philips CM120 Transmission Electron Microscope (TEM) operating at 100 kV with a LaB6 filament under low dose condition (less than 20 electrons/Å^2) at a nominal magnification of 60.000x on a Pelletier cooled slow scan CCD camera (Model 794, Gatan, Pleasanton) resulting in a pixel spacing of 2.8 Ǎ. A total of 5110 molecular images were selected interactively and analyzed using the IMAGIC software package [8] (Image Science Software, Berlin, Germany) as

described earlier [9]. Briefly original images were aligned and partitioned into classes corresponding to molecular views whose angular relationships were determined by the common line method (van heel angular reconstruction). The resulting 3-D model showed a two-fold symmetry axis that was imposed during subsequent refinements. The Fourier Shell Correlation Function obtained by comparing two distinct PFV integrase reconstructions obtained by splitting the data set in two indicated a resolution of 28 Ǎ using the 0.5 FSC criterion.

Supplementary References:

1. Kask P, Palo K, Ullmann D, Gall K (1999) Fluorescence-intensity distribution analysis and its application in biomolecular detection technology. ProcNatlAcadSciUSA 96: 13756-13761.
2. Beechem J, Gratton E, Ameloot M, Knuston J, Brand L (1991) The global analysis of fluorescence intensity andanisotropy decay data: second-generation theory and programs. Topics in fluorescence spectroscopy. New York: Plenum: J L, editor. pp. 241-305.
3. Marquardt DW (1963) An Algorithm for Least-Squares Estimation of Non-Linear Parameters. SIAM J Appl Math: 431-441.
4. Didier P, Godet J, Mely Y (2009) Two-photon two-focus fluorescence correlation spectroscopy with a tunable distance between the excitation volumes. JFluoresc 19: 561-565.
5. Perrin F (1936) Mouvement brownien d'un ellipsoide (II). Rotation libre et depolarisation des fluorescences. Translation et diffusion de molecules ellipsoidales. J Phys Radium Ser 7: 1-11.
6. Egelé C, Piémont E, Didier P, Ficheux D, Roques B, et al. (2007) The single-finger nucleocapsid protein of moloney murine leukemia virus binds and destabilizes the TAR sequences of HIV-1 but does not promote efficiently their annealing. Biochemistry 46: 14650-14662.
7. Merkel G, Andrake MD, Ramcharan J, Skalka AM (2009) Oligonucleotide-based assays for integrase activity. Methods 47: 243-248.
8. Van Heel M, Harauz G, Orlova EV, Schmidt R, Schatz M (1996) A new generation of the IMAGIC image processing system. JStructBiol 116: 17-24.
9. Jawhari A, Uhring M, De CS, Crucifix C, Tocchini-Valentini G, et al. (2006) Structure and oligomeric state of human transcription factor TFIIE. EMBO Rep 7: 500-505.
10. Michel F, Crucifix C, Granger F, Eiler S, Mouscadet JF, et al. (2009) Structural basis for HIV-1 DNA integration in the human genome, role of the LEDGF/P75 cofactor. EMBO J 28: 980-991.
11. Ludtke SJ, Baldwin PR, Chiu W (1999) EMAN: semiautomated software for high-resolution single-particle reconstructions. JStructBiol 128: 82-97.
12. Rosenthal PB, Henderson R (2003) Optimal determination of particle orientation, absolute hand, and contrast loss in single-particle electron cryomicroscopy. JMolBiol 333: 721-745.
